# Surface attachment, promoted by the actomyosin system of *Toxoplasma gondii* is important for efficient gliding motility and invasion

**DOI:** 10.1186/s12915-016-0343-5

**Published:** 2017-01-18

**Authors:** Jamie A. Whitelaw, Fernanda Latorre-Barragan, Simon Gras, Gurman S. Pall, Jacqueline M. Leung, Aoife Heaslip, Saskia Egarter, Nicole Andenmatten, Shane R. Nelson, David M. Warshaw, Gary E. Ward, Markus Meissner

**Affiliations:** 1 Wellcome Trust Centre For Molecular Parasitology, Institute of Infection, Immunity & Inflammation, Glasgow Biomedical Research Centre, University of Glasgow, 120 University Place, Glasgow, G12 8TA UK; 2 Department of Biology, Indiana University, Bloomington, Myers Hall 240, 915 E 3rd St Bloomington, Bloomington, IN 47405 USA; 3 University of Vermont, Department of Molecular Physiology and Biophysics Burlington, Vermont, 05405 USA; 4 University of Vermont, Department of Microbiology and Molecular Genetics, College of Medicine, Burlington, VT 05405 USA

**Keywords:** Actin, Myosin, Motility, Attachment, *Toxoplasma*, Apicomplexa, Host cell invasion, Membrane flow

## Abstract

**Background:**

Apicomplexan parasites employ a unique form of movement, termed gliding motility, in order to invade the host cell. This movement depends on the parasite’s actomyosin system, which is thought to generate the force during gliding. However, recent evidence questions the exact molecular role of this system, since mutants for core components of the gliding machinery, such as parasite actin or subunits of the MyoA-motor complex (the glideosome), remain motile and invasive, albeit at significantly reduced efficiencies. While compensatory mechanisms and unusual polymerisation kinetics of parasite actin have been evoked to explain these findings, the actomyosin system could also play a role distinct from force production during parasite movement.

**Results:**

In this study, we compared the phenotypes of different mutants for core components of the actomyosin system in *Toxoplasma gondii* to decipher their exact role during gliding motility and invasion. We found that, while some phenotypes (apicoplast segregation, host cell egress, dense granule motility) appeared early after induction of the *act1* knockout and went to completion, a small percentage of the parasites remained capable of motility and invasion well past the point at which actin levels were undetectable. Those *act1* conditional knockout (cKO) and *mlc1* cKO that continue to move in 3D do so at speeds similar to wildtype parasites. However, these mutants are virtually unable to attach to a collagen-coated substrate under flow conditions, indicating an important role for the actomyosin system of *T. gondii* in the formation of attachment sites.

**Conclusion:**

We demonstrate that parasite actin is essential during the lytic cycle and cannot be compensated by other molecules. Our data suggest a conventional polymerisation mechanism in vivo that depends on a critical concentration of G-actin. Importantly, we demonstrate that the actomyosin system of the parasite functions in attachment to the surface substrate, and not necessarily as force generator.

**Electronic supplementary material:**

The online version of this article (doi:10.1186/s12915-016-0343-5) contains supplementary material, which is available to authorized users.

## Background

Apicomplexan parasites employ a unique form of movement, termed gliding motility, to disseminate and invade host cells. The current model for gliding motility is centred on the actomyosin system of the parasite in force generation during this process. According to the ‘linear motor’ model, micronemal transmembrane proteins, such as MIC2, are secreted at the apical pole of the parasite and act as force transmitters by interacting with the substrate surface and the actomyosin system, similar to the role of integrins in other motility systems [1]. Accordingly, this model predicts a smooth actomyosin dependent rearwards translocation of the microneme-substrate complex, which results in forward gliding on the substrate or into the host cell [2]. However, recent reverse genetic approaches demonstrated that parasites remain motile in the absence of what were thought to be key components of the motility machinery, including parasite actin [3–6]. In an attempt to reconcile these data, we proposed a novel, hypothetical model based on gelation/solation [3], where actin/myosin is required for force transmission rather than force production [1]. This model predicts the generation of an osmotic gradient that results in membrane tension and retrograde membrane flow. In order to regulate membrane balance, the parasite then either sheds membrane at the posterior end, as previously observed [7], and/or recycles the membrane via endocytosis, as suggested for other systems [8, 9].

Many other eukaryotic cells move by crawling, a process powered by the controlled assembly and disassembly of the actin cytoskeleton in proximity to the plasma membrane [10, 11]. While it was previously assumed that the majority of the force for the retraction of the cell rear is powered by myosins, several alternative mechanisms can operate within the cell that also power motility [10, 11]. For example, the motility of *Caenorhabditis elegans* sperm occurs in the absence of any actin and therefore myosin activity [12]; instead, force is generated by controlled polymerisation of the major sperm protein. Furthermore, when tumour cells are confined within a microenvironment, motility depends on an osmotic engine and can be independent of actomyosin activity [13]. Finally, migrating cells can employ various migration modes in response to their microenvironment [14], which allows them to move even in the absence of adhesive coupling [15]. For example, crawling cells, such as *Dictyostelium* and human leukocytes, can swim efficiently when suspended in a viscous medium [16, 17], demonstrating that adhesion to a solid substrate is not always necessary for movement [18]. It is thus possible that motility can be driven purely by surface membrane flow, which itself results from a secretory-endocytic cycle acting as a fluid drive from the anterior to the posterior end of the cell [18]. In support of membrane flow, many motile cells show a distinct capping activity of surface ligands [8]. Furthermore, surface capping depends on vesicular transport, and both on actin and microtubule function [18]. In good agreement with these observations, numerous modulators of endocytic and secretory trafficking have been demonstrated to be key regulators of cell motility [19], leading to the hypothesis that membrane transport and retrograde flow during motility are rate-limiting for cell forward translocation [20].

In the case of apicomplexan parasites, a recent study demonstrated that retrograde membrane flow of malaria sporozoites occurs even at relatively high concentrations of the actin-disrupting drug Cytochalasin D (CD) [21]. Furthermore, biophysical studies on malaria sporozoites have demonstrated the discrete, localised turnover of attachment sites that are not evenly translocated along the surface of the parasite [22]. This involves the formation/disengagement of adhesion sites at the front and rear ends of the zoite, while the sporozoite undergoes a stretching phase. Interestingly, actin is important for the definition and release of the attachment sites [23], raising the possibility that apicomplexan motility may be similar to amoeboid-like crawling.

In *T. gondii*, an alternative explanation for the observed phenotypes for mutants of the actomyosin system is the possibility of functional redundancy of actomyosin components [24]. While redundancies cannot be ruled out, in particular for myosins and micronemal proteins [3, 4], even the removal of ostensibly critical structural proteins does not completely block gliding motility or host cell invasion. For example, removal of the gliding-associated protein GAP45 results in the relocation of the remaining MyoA-complex components to the cytosol and severe disruption of the inner membrane complex (IMC), within which the motor complex is normally anchored [3, 25]. While these *gap45* conditional knockout (cKO) parasites are completely blocked in host cell egress, they remain motile and can invade host cells [3]. These surprising findings have led to a reassessment of the mechanisms underlying parasite motility and host-cell invasion, as recently reviewed [26]. In fact, not only do the roles of the individual components need to be reanalysed, but also their orientation and organisation within the parasite [26].

Here, we show that, during motility, the actomyosin system of the parasite functions in attachment to the surface substrate, and may, therefore, act as force transmitter rather than a force generator. We provide evidence that retrograde membrane flow, which may play a role in force generation and motility, does not depend on an intact actomyosin system.

## Results

### Actin is undetectable within 96 h of removal of *act1* in *act1* cKO

We previously reported that ACT1 is undetectable in *act1* cKO parasites 72 h after rapamycin-induced removal of *act1* [3]. A subsequent study suggested that residual actin may, in fact, be present in some of these parasites as long as 5 days after rapamycin treatment [27]. This led us to re-address the question of residual actin levels in the *act1* cKO parasites, using additional antibodies and additional controls.

**Fig. 1:**
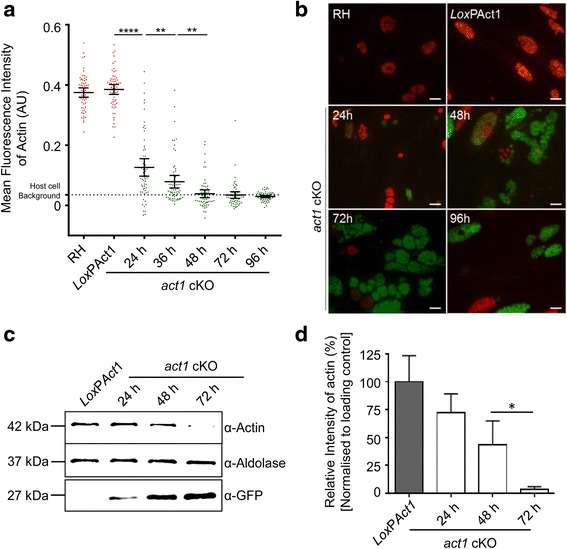
Actin is almost undetectable as soon as 72 h after rapamycin induction of *act1* excision. **a** Quantitative immunofluorescence assay (IFA) of actin. Vacuoles were stained with α-ACT1 (Soldati) at 0, 24, 48, 72 and 96 h post rapamycin induction. Fluorescence intensity was analysed using CellProfiler software. The internal background was calculated for each vacuole and was then subtracted from the calculated intensity and plotted with mean and 95% confidence intervals. *Dotted line* shows the host cell background calculated using a *YFP* ^+^ parasite strain without antibodies. *n* = 60 vacuoles analysed per time point. The datasets were compared with a two-tailed Student’s t-test. **** *P* < 0.0001, ** *P* < 0.01. **b** Representative images of the labelled vacuoles used to analyse the ACT1 levels over time. Images shown were processed together under the same conditions to highlight the ACT1 signal intensity in the vacuoles. After 72 h actin was undetectable in YFP(+) vacuoles by IFA. Scale bar: 10 μm. **c** Western blot analysis of actin protein level. Immunoblot made with parasite lysates taken at 0, 24, 48 and 72 h after rapamycin induction. Aldolase was used as a loading control. Expression of YFP upon *act1* excision was checked using α-GFP. **d** Relative levels of actin were analysed with LiCor Odyssey Image Studio 5.0 and normalised first using aldolase loading control and then compared against the LoxPAct1 control. The dataset was compared with a two-tailed Student’s t-test. Error bars represent standard deviation. * *P* < 0.05, *n* = 4

We began by comparing the specificity of different antibodies, from various labs, raised against apicomplexan actin (Additional file 1: Figure S1). While immunoblot analysis suggested that all antibodies are highly specific for *T. gondii* actin without recognising host cell actin (Additional file 1: Figure S1A), some of the tested antibodies demonstrated significant cross-reactivity within the parasites when used for immunofluorescence analysis, as previously reported [4]. We performed IFAs at 4 days (96 h) and 8 days (192 h) after rapamycin induction (Fig. 1b, c and [4]). Notably, we found that the polyclonal antibodies employed previously to quantify actin levels in the *act1* cKO parasites [27] showed a strong and persistent signal in proximity to the IMC of the parasite. This signal remained unchanged between 4 and 8 days after rapamycin treatment, even though the parasites are no longer expressing levels of actin above background within 4 days of induction (see below). Since this apparent cross-reactivity would interfere with accurate quantification of actin levels, we focused on two other antibodies that were highly specific and showed no cross-reactivity (Additional file 1: Figure S1).

Consistent with other studies, we found that actin levels in wildtype (wt) parasites were somewhat variable (Fig. 1a, b). Similar results were obtained with other housekeeping genes such as myosin light chain 1 (MLC1, Fig. 5a) and aldolase (not shown). Importantly, no overlap of actin levels could be observed between controls and *act1* cKO parasites as early as 48 h after rapamycin-induced excision of *act1* (Fig. 1a, b). At earlier time points (24–36 h) post induction, significant amounts of ACT1 were still detectable and protein levels were highly variable between vacuoles (Fig. 1a, b). Nevertheless, ACT1 levels were significantly reduced in comparison to control vacuoles. Importantly, 96 h after rapamycin induction, no ACT1 was detectable in YFP^+^ parasites. Lack of ACT1 detection by IFA correlates well with a recent study suggesting that no F-actin can be formed approximately 48 h after the removal of *act1* [28], the inference being that G-actin levels fall below the critical concentration required for polymerisation.

In good agreement with this result, quantitative immunoblotting of *act1* cKO parasites at different time points post-induction demonstrated the loss of all or nearly all of the ACT1 as early as 72 h post induction (Fig. 1c, d), confirming previous results [3].

In summary, our quantification demonstrates that actin is depleted in the *act1* cKO parasites between 72 and 96 h post induction. Based on these observations, assays were performed in a time course up to 96 h after addition of rapamycin to adequately readdress the effects of ACT1 loss.

### Loss of actin results in deleterious phenotypes for apicoplast maintenance and host cell egress but not gliding motility and host cell invasion

**Fig. 2:**
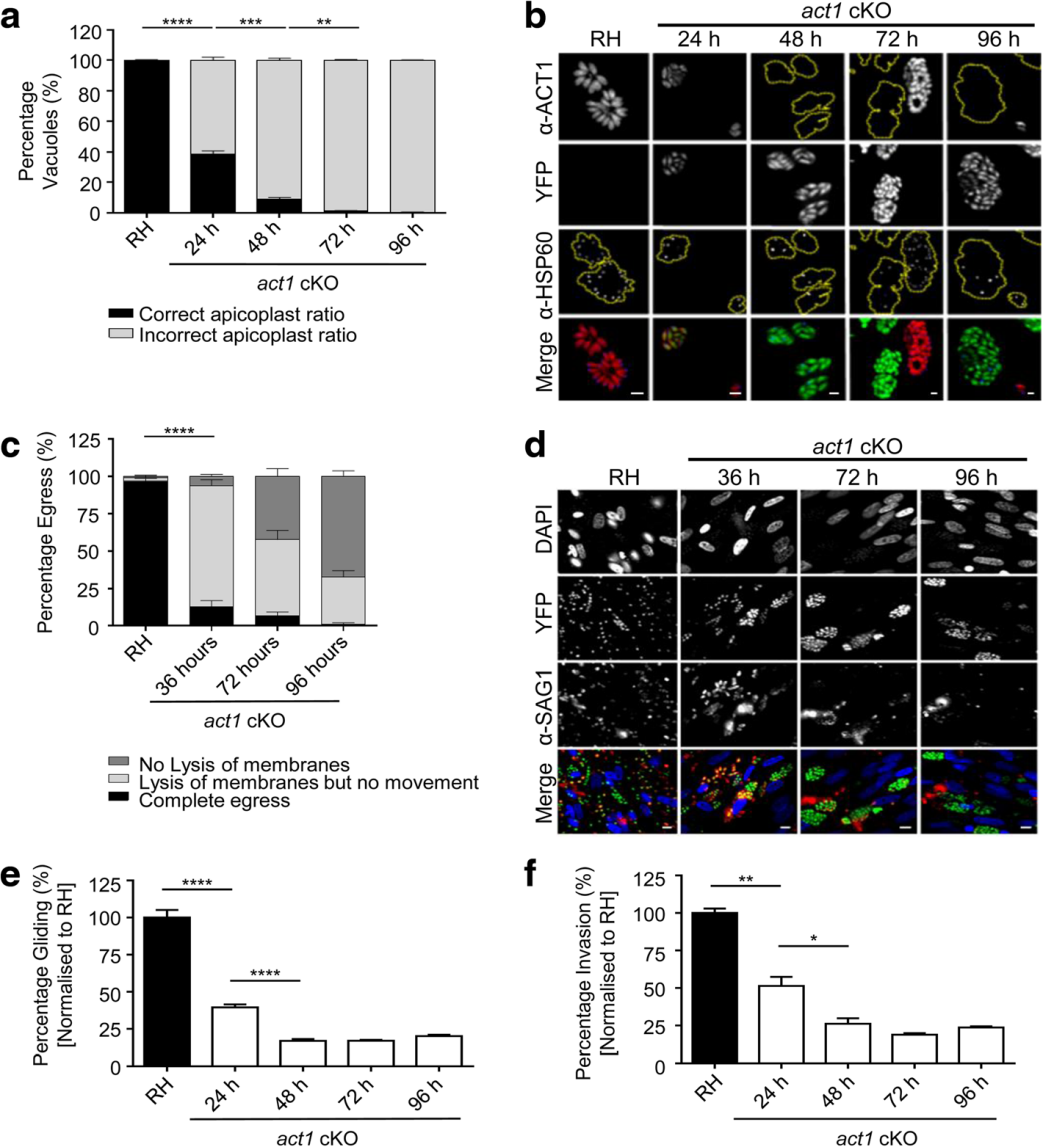
Phenotypic characterisation of the *act1* conditional knockout (cKO) at various time points after rapamycin-induced gene excision. **a** Quantification of apicoplast loss in the *act1* cKO over a time range. Loss of the apicoplast was quantified at 0, 24, 48 and 96 h after induction. Vacuoles were stained with α-ACT1 and α-HSP60 and scored for the correct ratio of apicoplasts per parasite. The apicoplast is lost in > 50% of the *act1* cKO parasites as early as 24 h after rapamycin induction. Error bars represent ± standard error of the mean (SEM). **b** Representative images of apicoplast loss. *Yellow dotted lines* indicate the edge of the vacuoles in greyscaled images. Apicoplast loss occurs even when there is still ACT1 present (24 h). The apicoplast was stained with an antibody against HSP60 [62]. **c** Egress in the *act1* cKO at different time points (0, 36, 72 and 96 h). Vacuoles were stained with α-SAG1 under non-permeabilising conditions and scored. Three conditions were considered: lysed and moved out of the vacuole, lysis of the membranes without the release of parasites, and no lysis of the parasitophorous vacuole membrane or host membrane. Error bars represent ± SEM. **d** Representative images of egress; at 72–96 h post induction, the *act1* cKO parasites are unable to lyse the membranes, as determined by positive SAG1 staining without prior permeabilisation and are not released from the vacuoles. Scale bars: 10 μm. **e** and **f** Trail deposition and invasion assays for the *act1* cKO compared to wt (RH) at time points 0, 24, 48 and 96 h after induction. After 48 h post induction, no significant change of trail deposition or invasion were observed, Error bars represent ± standard error of the mean. All experiments were performed in biological triplicate and compared with a two-tailed Student’s t-test, **** *P* < 0.0001, *** *P* < 0.001, ** *P* < 0.01, * *P* < 0.05

**Fig. 3:**
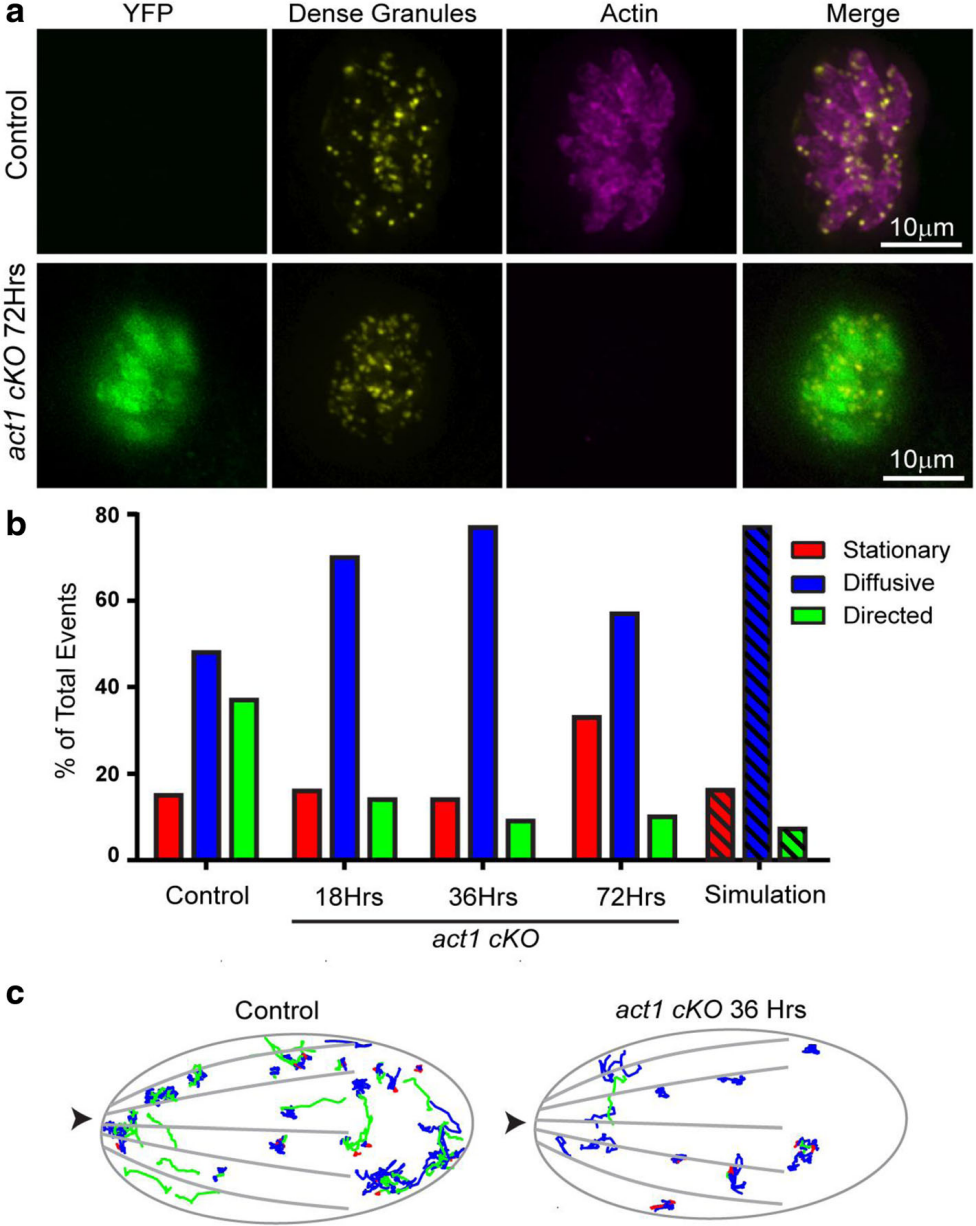
Loss of TgACT1 perturbs directed dense granule motions. **a** Fluorescence images of control LoxPAct1 parasites and LoxPAct1 parasites 72 h after rapamycin treatment. YFP (*green*); SAG1-∆GPI-mCherry to identify dense granules (*yellow*); anti-actin (*pink*). **b** Bar chart showing the percentage of granules exhibiting stationary (*red*), diffusive-like (*blue*) and directed (*green*) motion in control parasites and at 18, 36 and 72 h after rapamycin treatment. *Hashed bars* indicated the percentage of trajectories in each population when simulated pure 2D diffusion was analysed using the changepoint algorithm. **c** Parasite outline with dense granule trajectories overlaid from 10 control and 10 rapamycin treated parasites. *Arrowhead* indicates the parasites apical end

We previously demonstrated that parasite actin is required for apicoplast maintenance and host cell egress [3]. These phenotypes were followed over time after *act1* excision to estimate the time when G-actin levels are below the critical concentration in order to form F-actin. Significant numbers of *act1* cKO parasites started to lose their apicoplasts as early as 24 h post-induction, indicating that once G-actin levels drop below a critical concentration, the apicoplast cannot be maintained during parasite replication (Fig. 2a, b). By 96 h after rapamycin induction of *act1*, we were unable to see any parasitophorous vacuoles that had a normal number of apicoplasts (Fig. 3b). Similar to apicoplast maintenance, host cell egress is significantly decreased as soon as 36 h post induction and was completely blocked 96 h post induction (Fig. 2c, d).

Next, we analysed the capability of *act1* cKO parasites to move by gliding motility and to invade the host cell at different time points after rapamycin induction (Fig. 2e, f). In good agreement with previous results [3], overall motility and host cell invasion were significantly reduced in the *act1* cKO parasites, with less than approximately 25% being motile and capable of invading host cells compared to control parasites (Fig. 2e, f). This phenotype was observed as early as 48 h after rapamycin induction, at a time point when significant amounts of actin were still detectable (Fig. 1). Interestingly, and in striking contrast to the results with apicoplast inheritance and egress, gliding and invasion rates remained constant at 48–96 h post induction, suggesting that once actin levels fall below a critical concentration, these phenotypes are not further affected. The simplest explanation for this result is that apicoplast maintenance and egress are completely dependent on actin, whereas gliding and invasion can occur independent of actin, albeit at significantly reduced efficiencies.

### A critical concentration of actin is required to sustain dense granule motility

A recent study demonstrated the essential role of actin and the unconventional myosin F (MyoF) in directed dense granule transport [29]. To assess if potential, residual F-actin in *act1* cKO parasites is capable of serving as a track for MyoF-dependent dense granule transport, dense granule motions were tracked at various times after actin excision. In good agreement with the results obtained for apicoplast replication, parasite egress, parasite gliding and invasion, we observed an approximately 50% reduction in the percentage of granules moving in a directed manner from 37% in control to 14% as early as 18 h post excision (Fig. 3a, b). At 36 and 72 h post excision, directed granule motion was completely ablated (Fig. 3b, c) given that the percentage of trajectories in each population was indistinguishable from simulated pure 2D diffusion analysed using the changepoint algorithm (See Methods for details) (Fig. 3b; hashed bars).

The observation of diminished granule movement soon after induction provides further evidence that apicomplexan actin in vivo requires a critical monomer concentration (G-actin) in order to form sufficient F-actin to serve as a substrate for myosin motors such as MyoF. We therefore conclude that *act1* cKO parasites are a powerful tool to analyse actin-independent motility and invasion processes.

### Conventional actin disrupting drugs are not exclusively specific for *Toxoplasma* actin

Intriguingly, the phenotypes described above differ significantly from the effects caused by incubating parasites in the presence of drugs used to modulate F-actin turnover, such as CD or Latrunculins. For example, treatment of intracellular parasites with CD results in the formation of huge residual bodies, where organelles such as the apicoplast or secretory organelles accumulate [30, 31], which is not observed in the case of the *act1* cKO parasites [3].

**Fig. 4:**
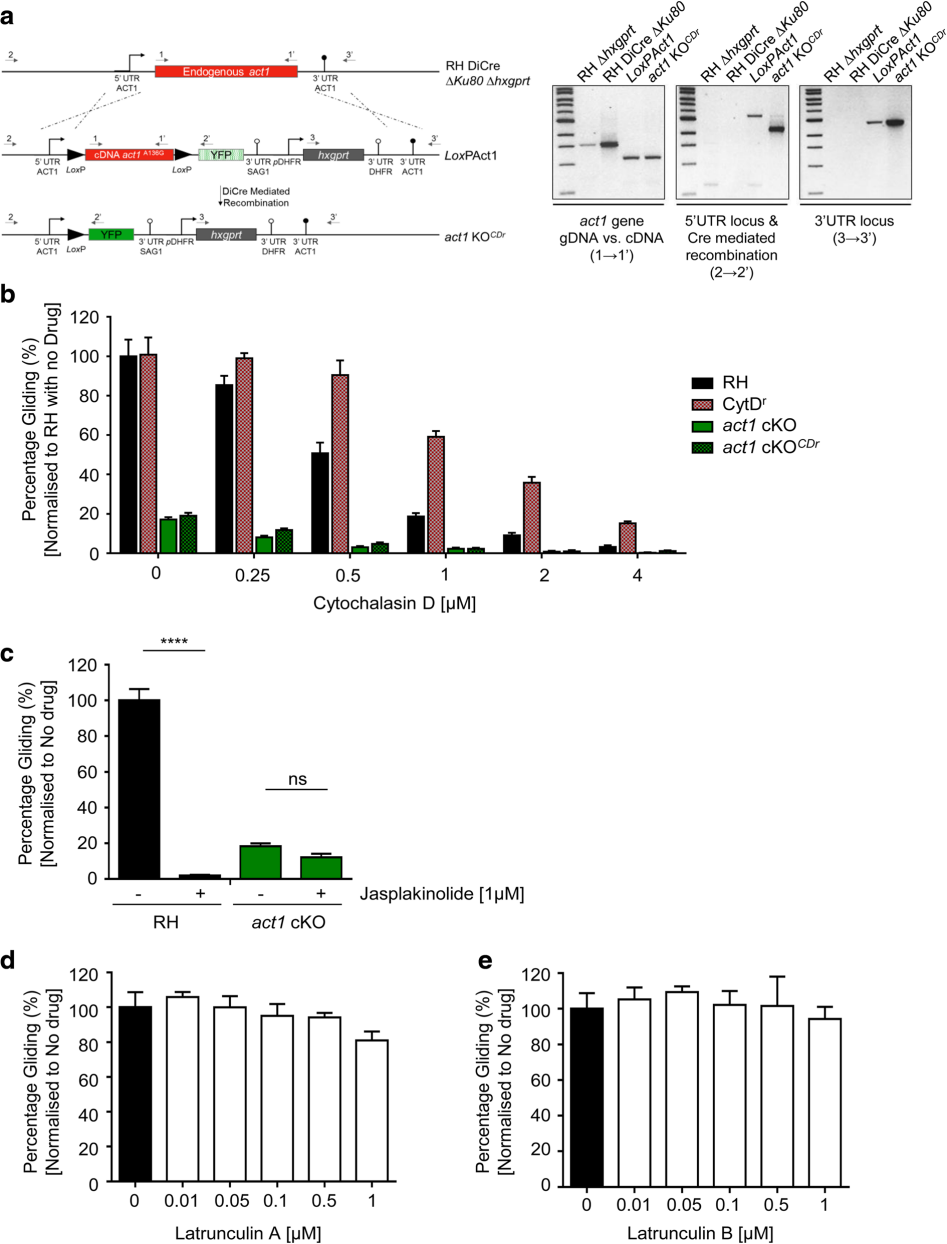
Conventional actin disrupting drugs are not exclusively specific for *Toxoplasma* actin. Evaluation of the off-target effect of cytochalasin D (CD). **a** Generation of an inducible CD resistant actin strain (*act1* cKO^*CDr*^). Schematic of the inducible CD resistant *act1* conditional knockout (cKO) geneswap vector, encoding the *act1* resistance mutation for CD (A136G), flanked by LoxP sites with a reporter cassette of YFP. Analytical PCR checked integration with primer sets highlighted in the schematic. **b** Trail deposition assay for RH, CytD^r^, *act1* cKO and *act1* cKO^CDr^ treated with increasing concentrations of CD (0–4 μM). No significant difference in gliding rates was observed between the *act1* cKO or *act1* cKO^CDr^. The CytD^r^ parasites are inhibited in gliding motility at high concentrations of CD (>1 μM). Error bars represent ± standard error of the mean (SEM). **c** Trail deposition assay of RH parasites and *act1* cKO parasites in 1 μM Jas. Forcing polymerisation blocks motility in wildtype cells but has no additional effect on *act1* cKO. Error bars represent ± SEM. **d**, **e** Latrunculins have no effect on parasite motility. Parasites treated with increasing concentrations of either latrunculin A (**d**) or latrunculin B (**e**). Error bars represent ± SEM. All experiments were performed in biological triplicate and the datasets were compared with a two-tailed Student’s t-test, **** *P* < 0.0001, non-significance (ns) *P* >0.05

Our finding that parasites remain motile in the absence of ACT1 further suggests that CD might act on an unknown target, a hypothesis supported by an earlier study in which CD resistant parasites were isolated that do not have any mutations in *TgAct1* [32]. To test this hypothesis, we generated a parasite line where endogenous actin has been replaced with a LoxP-flanked copy of *act1* containing a mutation (Ala136Gly) that confers CD resistance [32] and can be excised in a DiCre-dependent manner (Fig. 4a). Consequently, these parasites only express CD resistant actin before removal of *act1* ^*CDr*^. We compared the CD sensitivity of this new parasite line, called *act1* ^*CDr*^ cKO, to that of control (RH) parasites, CytD^r^ parasites [32] and the *act1* cKO parasites. Interestingly, the gliding rates of all parasite lines, including CytD^r^ parasites, were significantly reduced at higher concentrations of CD (1–4 μM) and it appeared the ACT1-specific effect was limited to ≤ 0.5 μM CD (Fig. 4b). The observed reduction in motility of CytD^r^ parasites is consistent with a previous study, demonstrating loss of motility in the presence of 1 μM CD [33]. Importantly, in the absence of ACT1 or ACT1^CDr^, parasites remained sensitive to CD (Fig. 4b); in both cases, the gliding rate was reduced from approximately 20% in untreated parasites to 1% in the presence of high CD concentrations, indicating that CD was acting on (a) yet unidentified target(s) in a non-ACT1 specific manner in *T. gondii*. Conclusions drawn from experiments using high concentrations of CD should, therefore, be interpreted with caution. Next, we tested the parasites’ ability to glide in the presence of the F-actin polymerisation drug Jasplakinolide (Jas). Jas was previously found to block attachment and motility of *P. berghei* sporozoites [34]. At lower concentrations, *T. gondii* tachyzoites are inhibited in both circular and helical gliding but exhibit a counter-clockwise twirling motion [35]. In agreement with previous findings, we found that forcing polymerisation blocks motility in wt parasites, whereas no significant reduction is observed for the *act1* cKO (Fig. 4c). We also tested latrunculins A and B, independent F-actin disrupting drugs that have been previously described to act on apicomplexan actin [36]. Surprisingly, even at 1 μM, we were unable to observe any significant effect on gliding rates (Fig. 4d), demonstrating that latrunculins are not acting on *Toxoplasma* tachyzoite actin. This conclusion supports previous observations for *Plasmodium berghei* gliding motility [37]. In addition, the PfACT1 crystal structure suggests that latrunculins should not effectively bind apicomplexan ACT1 [38].

In summary, the experiments presented above demonstrate that, in the absence of actin, less than 25% of parasites remain capable of gliding motility and host cell invasion and establish *act1* cKO parasites as a valuable tool for understanding the role of actin in the invasion and gliding mechanisms of *T. gondii*. The observed phenotype for the *act1* cKO parasites is very similar to the phenotype observed for a clonal knockout for *myoA* [3]. We therefore re-addressed the role of myosins during gliding motility.

### Expression levels of MyoC determine its localisation to the IMC in the *myoA* KO

It was previously suggested that MyoC can compensate for MyoA motor function in *myoA* KO parasites [3, 39]. In addition, it has been shown that MyoA and MyoC can interact with the same light chain, MLC1, and expression of MyoC from a heterologous promoter demonstrated a certain degree of MyoC association with the parasite pellicle in the absence of MyoA [24].

**Fig. 5:**
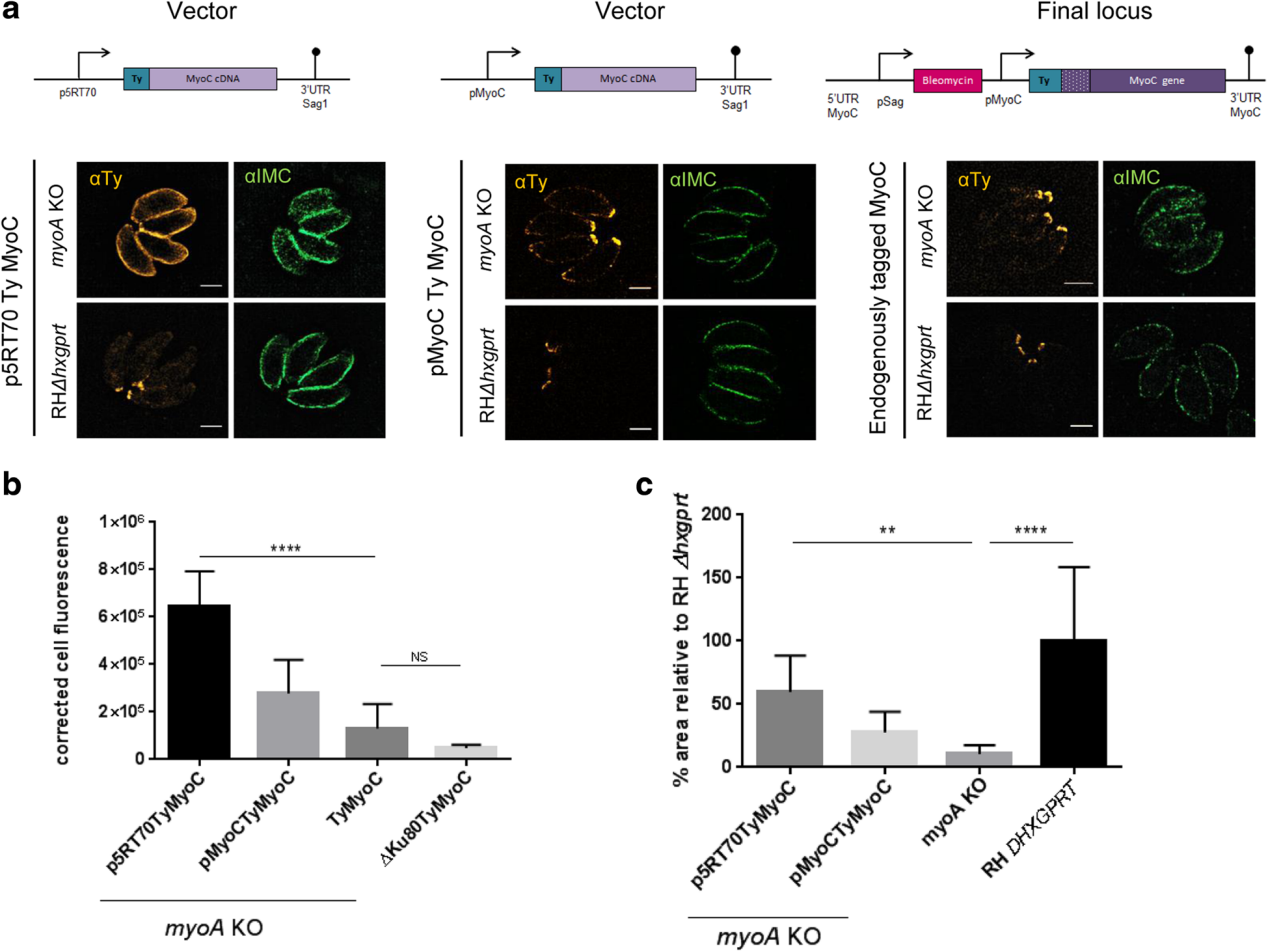
Expression levels of MyoC determine localisation to the inner membrane complex in the *myoA* knockout (KO) parasites. **a** Schematics of the plasmid (*upper panel*) and localisation of *Myo*C under different expression levels in both RH Δ*hxgprt* and *myoA KO* (*lower panel*). **a** Constitutive overexpression (p5RT70-Ty-MyoC; *left pane*l) and MyoC under its endogenous promoter (pMyoC-Ty-MyoC; *middle panel*), these constructs were randomly integrated. The endogenous MyoC locus was tagged in RH Δ*Ku80* via double homologous recombination (*right panel*). Schematic represents the final locus. Scale bar: 2 μm. **b** Corrected cell fluorescence intensities for different MyoC expression levels were measured using α-Ty. One-way ANOVA followed by Tukey’s post hoc test was used to compare means between groups. *** *P* < 0.0001, non-significance (ns) *P* > 0.05. **c** Plaque assay of *myoA* KO expressing the three different constructs. RH Δ*hxgprt* was used as the control. The plaque area was measured using ImageJ with error bars representing ± standard deviation (*n* = 15 plaques). One-way ANOVA followed by Tukey’s post hoc test was used to compare means between groups. **** *P* < 0.0001, ** *P* < 0.001

In order to address if the localisation of MyoC at the IMC in the *myoA* KO parasites depends on the level of MyoC expression, we expressed a second copy of TyMyoC under the control of either a strong promoter, p5RT70 [40], or its own promoter, pMyoC, and compared its localisation with that of an endogenously tagged version (Fig. 5a).

While strong expression of MyoC results in efficient relocalisation from the parasite basal end to the periphery of the *myoA* KO parasites (Fig. 5a, b), as previously reported [24], expression of MyoC under its own promoter or endogenously-tagged MyoC showed only slight relocalisation, with the majority of the protein being correctly localised to the basal ring of the parasites. This demonstrates that the localisation of MyoC to the IMC is critically dependent on strong expression levels of the protein, which are not reached with native MyoC expression levels in the *myoA* KO parasite (Fig. 5b). We next analysed propagation of these parasite strains using plaque assays and found that the relative plaque size is larger in *myoA* KO parasites strongly overexpressing MyoC compared to parasites expressing MyoC under the control of pMyoC (Fig. 5c). This demonstrates that overexpression of MyoC can partially complement the phenotype of the *myoA* KO parasites (Fig. 5c). Since the localisation of MyoC to the IMC is apparently critical for complementation of the *myoA* KO parasites, we decided to analyse *mlc1* cKO in more detail, given that MLC1 is thought to be required to anchor both MyoA and MyoC to the pellicle [3, 39].

### Parasites lacking MLC1 can be maintained in cell culture for several weeks

**Fig. 6:**
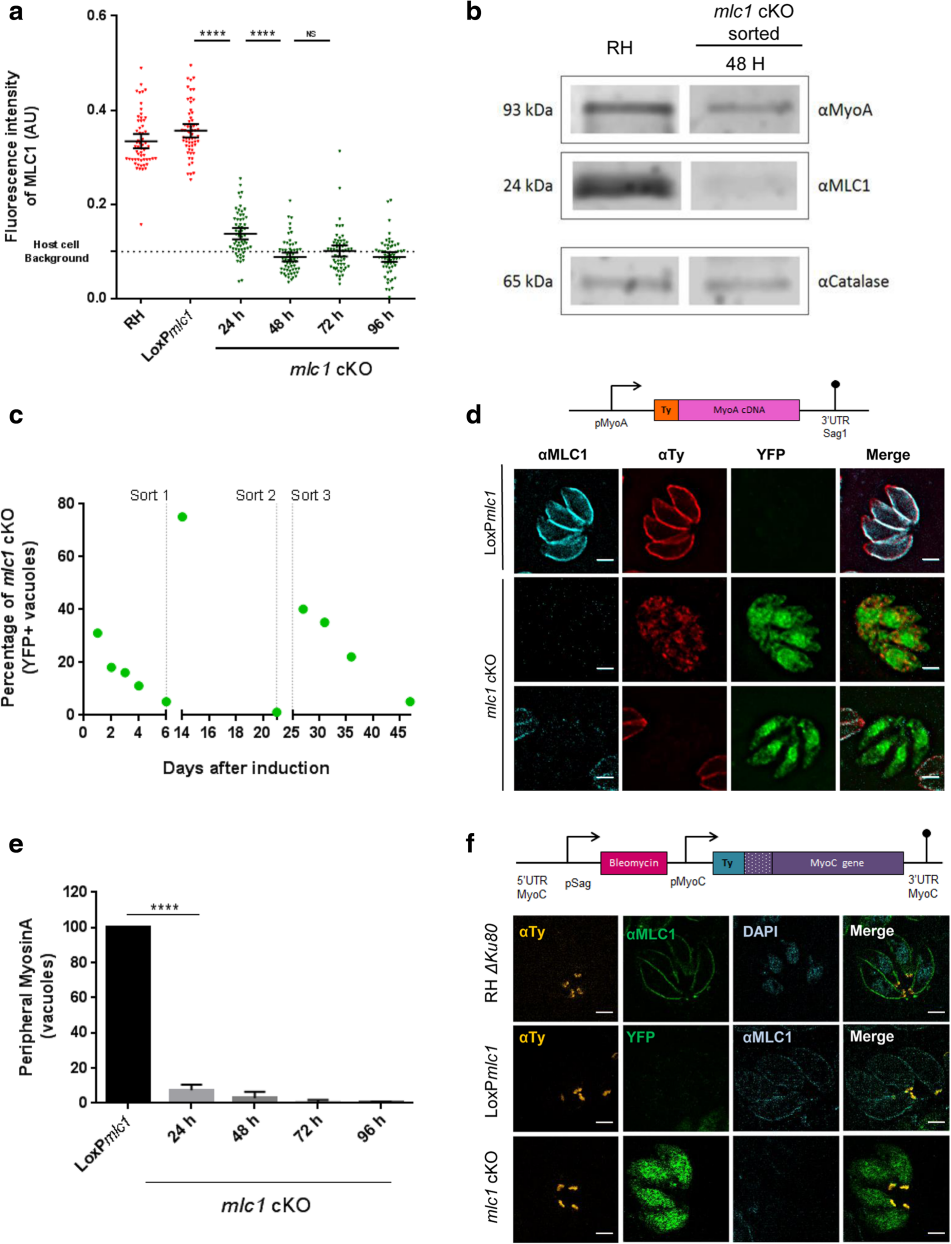
Parasites without MLC1 can be maintained in cell culture for several weeks. **a** MLC1 proteins levels were evaluated by measuring the fluorescence intensity of vacuoles stained with α-MLC1 at 0, 24, 48, 72 and 96 h post induction. Fluorescence intensity was analysed using CellProfiler software. The *lower dashed line* indicates the fluorescence background in the red channel obtained by using the YFP^+^ parasite strain without antibodies. *Bars* indicate mean with 95% confidence intervals. One-way ANOVA followed by Tukey’s post hoc test was used to compare means between groups. **** *P* < 0.0001, non-significance (ns) *P* > 0.05. **b** Western blot analysis of sorted *mlc1* conditional knockout (cKO) parasites to determine the protein levels of MyoA and MLC1. Catalase was used as loading control. **c** *mlc1* cKO culture maintenance. Long term viability of YFP+ *mlc1* cKO was assayed using FACs sorting. Six days after induction, parasites were sorted for YFP+ signal, which results from excision of the *mlc1* gene, and put back in culture. *Vertical dashed lines* indicate three sequential sortings over time of the maintained *mlc1* cKO culture. The population of *mlc1* cKO/YFP+ vacuoles was counted in 20 fields of view using 40× objective, and expressed as a percentage value over total number of vacuoles in different time points. **d** Depletion of MLC1 results in mislocalisation and absence of MyoA from the inner membrane complex. *Top panel*: Scheme of pMyoA-Ty-MyoA plasmid that was randomly integrated into either the RH Δ*hxgprt* or LoxP*mlc1* genome. MyoA cDNA was fused to a Ty tag under the pMyoA promoter. *Bottom panel*: Immunofluorescence analysis of Ty-tagged MyoA in RH Δ*hxgprt* and *mlc1* cKO parasites using α-Ty and α-MLC1 antibodies. **e** Percentage of *mlc1* cKO parasites expressing correctly localised MyoA using Ty-MyoA at 0, 24, 48 72 and 96 h after induction. Graphic shows mean ± standard deviation of three independent experiments. One-way ANOVA followed by Tukey’s post hoc test was used to compare means between groups. **** *P* < 0.0001. **f** Evaluation of MyoC localisation in the *mlc1* cKO. *Top panel*: scheme of the endogenous MyoC tagging plasmid. *Bottom panel*: Immunofluorescence analysis of Ty-tagged MyoC in RH Δ*hxgprt*, LoxP*mlc1* and *mlc1* cKO parasites. Scale bar represents 2 μm

We previously analysed the *mlc1* cKO 96 h after rapamycin induction; based on qualitative immunofluorescence analysis, no MLC1 was detectable at this time point [3]. To verify that MLC1 is depleted at 96 h, we quantified MLC1 protein levels by IFA, analogous to ACT1 quantification described above (Fig. 6a). We confirmed that, as early as 48 h after induction, no MLC1 can be detected in the cKO parasites; this was also confirmed using immunoblot analysis (Fig. 6a, b). We succeeded in keeping *mlc1* cKO parasites in cell culture for several weeks by mechanically releasing parasites and enriching the culture using FACS, demonstrating that MLC1 is essential for egress but dispensable for the following rounds of invasion and replication (Fig. 6c).

Next, we were interested in the localisation of MyoA and MyoC in *mlc1* cKO parasites. To determine this, we generated two independent lines for colocalization studies, one expressing Ty-tagged MyoA under the control of its endogenous promoter (pMyoA) and another with endogenously Ty-tagged MyoC (Fig. 6d–f). MyoA is completely undetectable at the parasite’s pellicle 72 h after rapamycin-induced excision of *mlc1* (Fig. 6d, e), demonstrating that MLC1 is crucial for the localization and stability of MyoA. In contrast, MyoC remains present and shows a normal localization at the basal end of the parasite in the absence of MLC1 (Fig. 6f).

In summary, we have generated and validated important parasite strains that can be used to analyse the role of the actomyosin system during gliding motility and host cell invasion and to investigate alternative mechanisms for these essential processes.

#### Kinetic analysis of actin and myosin independent motility and invasion

We previously demonstrated that conditional mutants for *act1*, *mlc1* and *myoA* invade the host cell via the tight junction, demonstrating that these parasites still use an active invasion mechanism and are not passively taken up by the host cell via phagocytosis [3]. However, kinetic analysis of *myoA* KO parasites demonstrated that both gliding speed and penetration times are significantly reduced, validating a direct function of MyoA during these processes. We were interested how *act1* cKO, *mlc1* cKO and *myoA* KO would compare during gliding motility and invasion.

To date, characterisation of gliding motility mainly depends on trail deposition assays, which allow a good estimate of overall motility rates. However, motility rates can be reduced by several factors, such as reduced or increased parasite attachment to the surface, and does not allow a firm conclusion regarding the function of a protein during this process. We speculated that parasites depleted in crucial parts of the actomyosin system should move significantly differently in terms of gliding speed and/or average distance travelled.

**Fig. 7:**
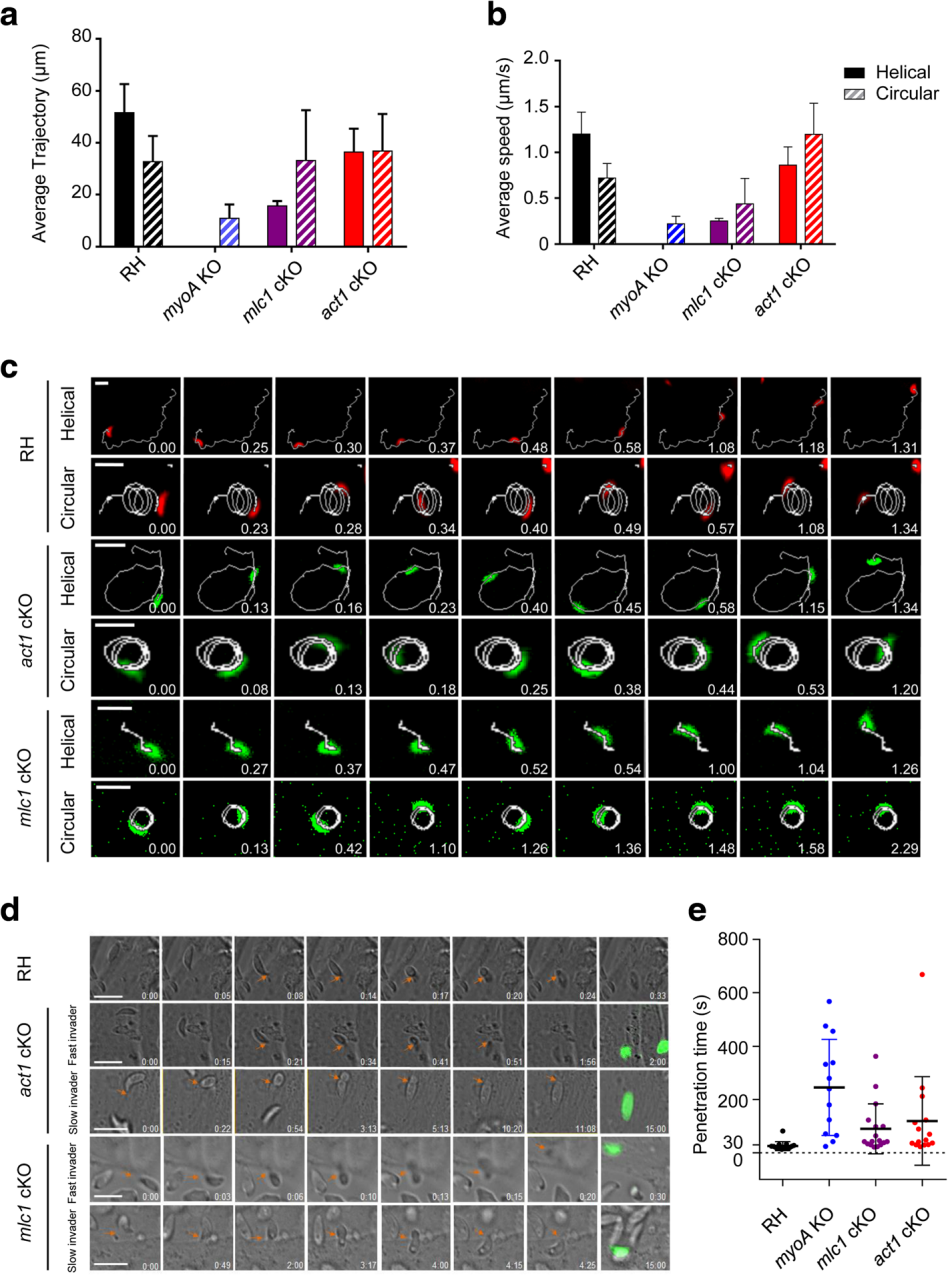
Kinetic analysis of 2D gliding and invasion of MyoA motor complex mutants. 2D analysis of gliding kinetics. **a** Graphic displays the average trajectory of 20 tracked parasites moving in helical (*solid pattern*) and circular fashion (*dashed pattern*). **b** Average speed of each motility boost of 20 tracked parasites for each strain. Trajectory, distance and time of moving parasites were tracked using the program ImageJ-wrMTrck. Values expressed are average ± standard deviation. **c** Image series show time lapse video microscopy of RH Δ*hxgprt*, *act1* conditional knockout (cKO) and *mlc1* cKO gliding kinetics. Circular and helical motions were recorded at one frame per second and analysed using the Fiji wrMTrck software. Software-generated tracks are overlaid in different times along parasites position. Scale bar represents 10 μm, numbers indicate time in minutes.seconds. **d** Image series showing real time invasion assays of RH Δ*hxgprt*, *act1* cKO and *mlc1* cKO. Real time invading parasites were recorded at one frame per second in DIC with a final image taken with FITC for the *act1* cKO and *mlc1* cKO to distinguish the YFP+ knockouts. *Orange arrows* indicate the tight junction from initial formation to closure. Scale bar represents 10 μm, numbers indicate time in minutes:seconds. **e** Penetration time of indicated strains determined by time lapse microscopy from tight junction formation to closure. Invasion events and time were tracked and calculated manually using the time stamper tool. Error bars represent ± standard deviation, *n* = 15 invasion events



Therefore, we performed time-lapse analysis of gliding parasites (Additional files 2–7: Movies S1–S6; Fig. 7a–c) to assess the ability of parasites to generate the necessary force for motility in the presence and absence of the actomyosin system of the parasite. We confirm that *myoA* KO parasites show significantly lower trajectory length (~7 μm, corresponding roughly to one parasite length) and an average speed of approximately 0.3 μm/sec, as reported previously [3]. Intriguingly, in the case of *act1* cKO and *mlc1* cKO parasites both average trajectory length and speed are increased compared to *myoA* KO parasites (Fig. 7a, b). While helical motility was significantly affected, with shorter average trajectories and reduced speed, circular gliding motility appeared relatively normal for *mlc1* cKO and *act1* cKO parasites (Fig. 7a–c). In good agreement with the gliding data, we found that host cell invasion can occur rapidly in *act1* cKO and *mlc1* cKO parasites, though several slow invasion events can be observed (Additional files 8–11: Movies S7–10; Fig. 7d, e). As is the case for gliding motility, the invasion of *myoA* KO parasites appears to be more strongly affected, with slower invasion events compared to *act1* cKO and *mlc1* cKO. This demonstrates that the parasite is capable of producing the force for motility and invasion in the absence of the actomyosin motor and suggests that actomyosin may instead be required to coordinate the force, most likely by regulating attachment sites, as observed in *Plasmodium* sporozoites [22, 34] and other diverse motility systems [41].

**Additional file 2:** **Movie S1.** Example of circular gliding motility of RH KillerRed. Time-lapse video microscopy of a RH KillerRed parasite gliding in a circular motion over a FBS-coated Ibidi glass bottom dish. Images were taken at one frame per second in A594 channel, DV Core. Movie is played at 20 frames per second. Movie supports Fig. 7c. (AVI 2799 kb)




**Additional file 3:** **Movie S2.** Example of helical gliding motility of RH KillerRed. Time-lapse video microscopy of a RH KillerRed parasite gliding in a helical motion over a FBS-coated Ibidi glass bottom dish. Images were taken at one frame per second in A594 channel, DV Core. Movie is played at 20 frames per second. Movie supports Fig. 7c. (AVI 1877 kb)




**Additional file 4:** **Movie S3.** Example of circular gliding motility of *mlc1* cKO. Time-lapse video microscopy of a parasite gliding in a circular motion over a FBS-coated Ibidi glass bottom dish. Images were taken at one frame per second in FITC channel, DV Core. Movie is played at 20 frames per second. Movie supports Fig. 7c. (AVI 2960 kb)




**Additional file 5:** **Movie S4.** Example of helical gliding motility of *mlc1* cKO. Time-lapse video microscopy of a parasite gliding in a helical motion over a FBS-coated Ibidi glass bottom dish. Images were taken at one frame per second in FITC channel, DV Core. Movie is played at 20 frames per second. Movie supports Fig. 7c. (AVI 2047 kb)




**Additional file 6:** **Movie S5.** Example of circular gliding motility of *act1* cKO. Time-lapse video microscopy of a parasite gliding in a circular motion over a FBS-coated Ibidi glass bottom dish. Images were taken at one frame per second in FITC channel, DV Core. Movie is played at 20 frames per second. Movie supports Fig. 7c. (AVI 1786 kb)




**Additional file 7:** **Movie S6.** Example of helical gliding motility of *act1* cKO. Time-lapse video microscopy of a parasite gliding in a helical motion over a FBS-coated Ibidi glass bottom dish. Images were taken at one frame per second in FITC channel, DV Core. Movie is played at 20 frames per second. Movie supports Fig. 7c. (AVI 1416 kb)




**Additional file 8:** **Movie S7.** Example of fast penetration of *act1* cKO. Time-lapse video microscopy of parasite invading an HFF cell. Images were taken at one frame per second in DIC channel, DV Core. Movie is played at 10 frames per second. Movie supports Fig. 7d. (AVI 3363 kb)




**Additional file 9:** **Movie S8.** Example of slow penetration of *act1* cKO. Time-lapse video microscopy of parasite invading an HFF cell. Images were taken at one frame per second in DIC channel, DV Core. Movie is played at 20 frames per second. Movie supports Fig. 7d. (AVI 1300 kb)




**Additional file 10:** **Movie S9.** Example of fast penetration of *mlc1* cKO. Time-lapse video microscopy of parasite invading an HFF cell. Images were taken at one frame per second in DIC channel, DV Core. Movie is played at 10 frames per second. Movie supports Fig. 7d. (AVI 2219 kb)




**Additional file 11:** **Movie S10.** Example of slow penetration of *mlc1* cKO. Time-lapse video microscopy of parasite invading an HFF cell. Images were taken at one frame per second in DIC channel, DV Core. Movie is played at 20 frames per second. Movie supports Fig. 7d. (AVI 2792 kb)


### Kinetics of 3D motility: MyoC cannot compensate for the loss of MyoA motor function

**Fig. 8:**
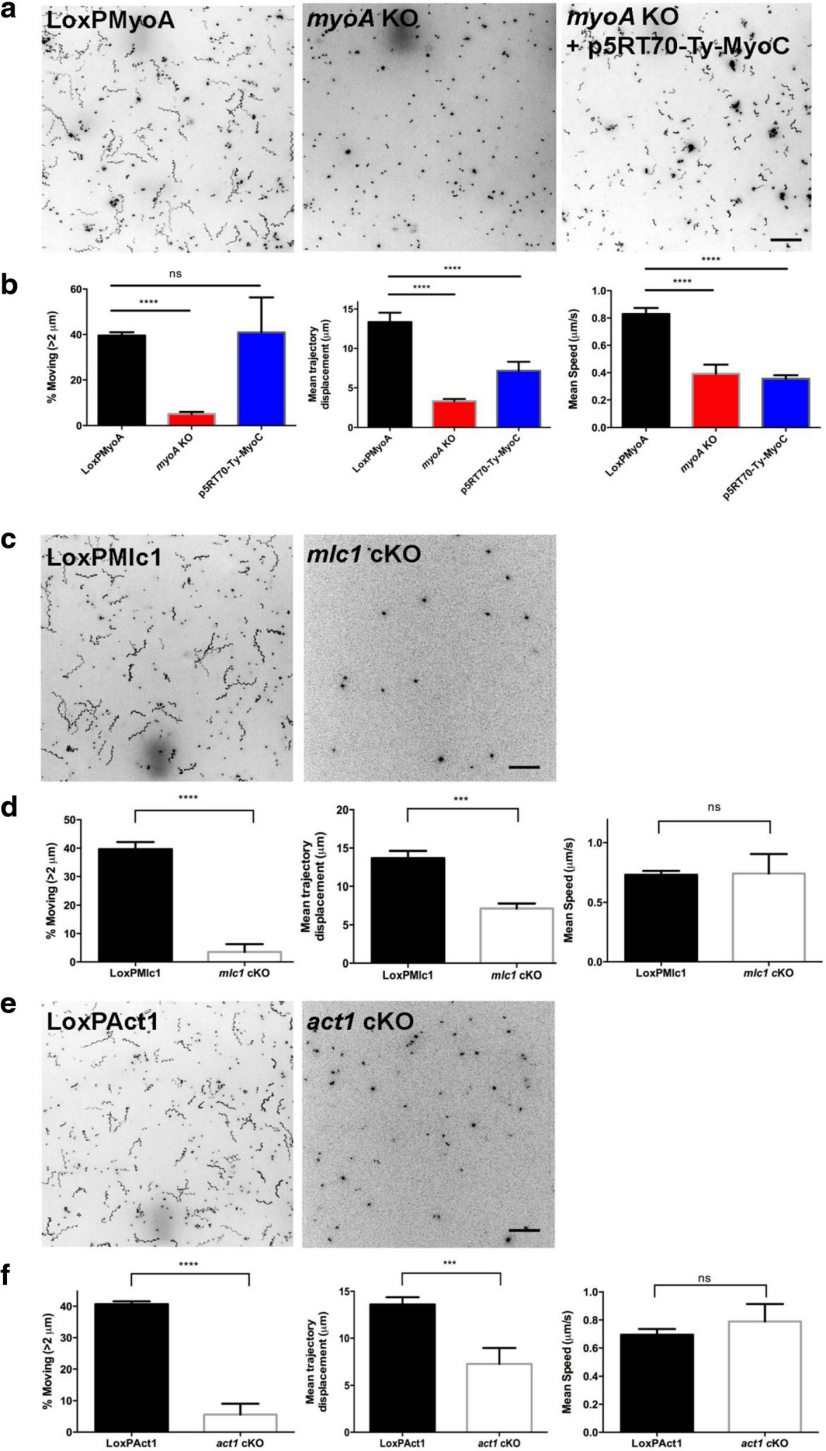
3D motility of the *myoA* knockout (KO), *mlc1* conditional knockout (cKO) and *act1* cKO parasites. **a** Maximum intensity projections (MIPs) for parental (LoxPMyoA), *myoA* KO and p5RT70-Ty-MyoC-expressing *myoA* KO parasites. **b** The percentage of total parasites moving (*left panel*), mean trajectory displacement (*middle panel*) and mean instantaneous speed (*right panel*) were significantly reduced for the *myoA* KO parasites (*red*). Of these motility parameters, p5RT70-Ty-MyoC (*blue*) restored the percentage of moving parasites back to wildtype levels (*black*), and partially restored the mean trajectory displacement, but was unable to complement the decrease in mean instantaneous speed. **c** MIPs for parental (LoxPMLC1) and *mlc1* cKO parasites. **d** The percentage of total parasites moving (*left panel*) and mean trajectory displacement (*middle panel*) were significantly reduced for the *mlc1* cKO parasites (*red*) but the mean instantaneous speed remained unaffected. **e** MIPs for parental (LoxPAct1) and *act1* cKO parasites. **f** The percentage of total parasites moving (*left panel*) and mean trajectory displacement (*middle panel*) were significantly reduced for the *act1* cKO parasites (*red*), but the mean instantaneous speed remained unaffected. Data shown are the results of three independent experiments (four for *mlc1* cKO), with each experiment performed in triplicate except for the induced KO samples (*mlc1* cKO and *act1* cKO), for which six technical replicates were performed per biological replicate due to low numbers of moving parasites. Datasets were compared by two-way ANOVA with Sidak’s multiple comparisons test. Error bars = SEM. The total number of parasites analysed was 2795 for LoxPMyoA, 3825 for *myoA* KO, 2910 for *myoA* KO + p5RT70-Ty-MyoC, 2443 for LoxPMlc1, 239 for *mlc1* cKO, 2435 for LoxPAct1, and 1037 for *act1* cKO. A 2-μm trajectory displacement cut-off was applied to exclude stationary parasites from calculations for mean trajectory speed and displacement (see Methods). Scale bar; 50 μm. *** *P* < 0.001, **** *P* < 0.0001, ns = not significant. The colour scheme for all MIPs was inverted for better visualisation of parasite trajectories

Next, we employed a Matrigel-based 3D motility assay to characterise the motility phenotypes of the mutants generated [42] under more physiological conditions. In good agreement with the time-lapse analysis of parasites gliding in 2D [3], a significantly decreased proportion of the *myoA* KO parasites was able to move in Matrigel compared to the parental line (39.5 ± 2.5% for LoxPMyoA parental, *n* = 2795 trajectories analysed vs. 5.1 ± 0.9% for *myoA* KO parasites, *n* = 3825 trajectories analysed) (Fig. 8a, b). Of the *myoA* KO parasites that were classified as motile, there was also a significant reduction in mean trajectory displacement and mean instantaneous speed compared to the parental line. Overexpression of Ty-tagged MyoC driven by the p5RT70 promoter in the *myoA* KO parasites was able to restore the percentage of moving parasites to wt levels and produce a modest increase in the mean trajectory displacement but not instantaneous speed (Fig. 8a, b; see also Additional file 12: Figure S2 for a comparison of the trajectory displacement distributions for the three lines). When we extended the analysis to examine *mlc1* cKO and *act1* cKO parasites, the results likewise closely mirrored those of the 2D assay (Fig. 7) – excision of *mlc1* or *act1* significantly reduced the percentage of moving parasites and mean trajectory displacement, but did not affect the mean instantaneous speed compared to their respective parental lines (Fig. 8c–f). Of note, it appears that mean trajectory displacement is more impacted in a 3D environment than in 2D, with few mutant parasites showing a displacement of more than one parasite length (Additional file 12: Figure S2), perhaps because they have to overcome more resistance in order to move in the Matrigel.

### The actomyosin system is required for surface attachment

**Fig. 9:**
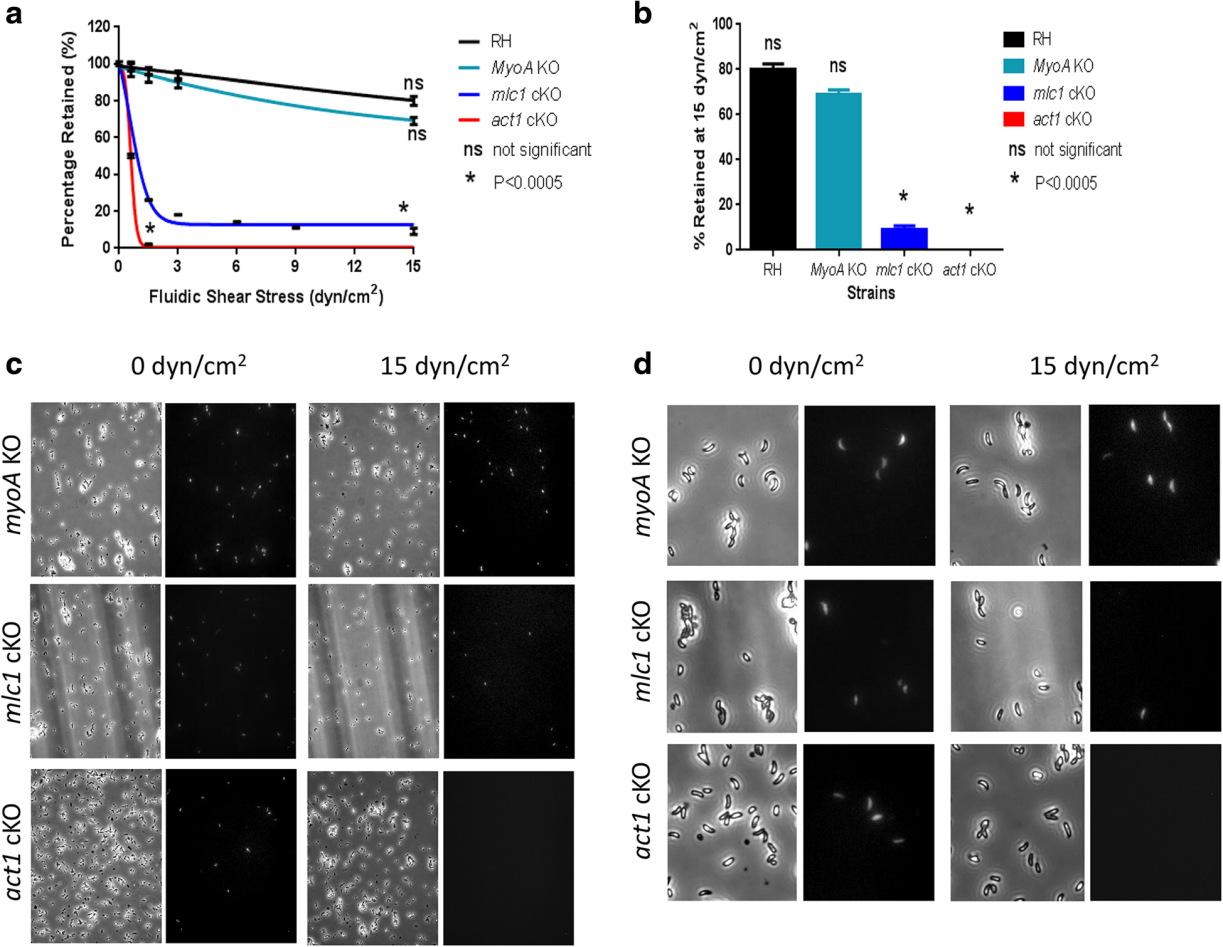
Attachment under fluidic shear stress. Evaluation of attachment under fluidic shear stress. **a** Percentage of parasites retained under fluidic stress relative to zero flow for RH, *myoA* knockout (KO), *mlc1* conditional knockout (cKO) and *act1* cKO strains. Data accumulated from *n* = 4 experiments for each strain, trendline ± standard deviation shown. **b** Bar graph showing relative percentage of parasites retained after 15 dyn/cm^2^ fluidic shear stress. The dataset was compared with a two-tailed Student’s t-test: ns, non-significant and * *P* < 0.0005. **c** Representative images at 0 and 15 dyn/cm^2^ fluidic shear stress. **d** Magnified inset of panel (**c**) showing individual parasites. Internal controls were used for comparison in each experiment. *myoA* KO: *myoA* KO non-fluorescent and GFP fluorescent RH control, *mlc1* cKO: *mlc* cKO YFP fluorescent and non-fluorescent RH control, *act1* cKO: *act1* cKO YFP fluorescent and non-fluorescent RH control. Scale bar 100 μm

To test the role of actin and the glideosome in attachment to surfaces, we employed flow chambers coated with collagen (Fig. 9). To this end, we tested the ability of the *myoA* KO, *mlc1* cKO and *act1* cKO parasites to withstand shear flow stress. While surface attachment of *myoA* KO parasites was similar to wt controls, both the *mlc1* cKO and *act1* cKO parasites were unable to promote efficient attachment to the surface and were removed even at very low shear flow rates (Fig. 9a–d). Only RH and the *myoA* KO can withstand shear forces up to 15 dyn/cm^2^, which is within the range of arterial shear stress [43]. These results suggest that the reason many *act1* cKO and *mlc1* cKO parasites cannot initiate gliding (Fig. 7c–f) is that they cannot attach sufficiently to the surface. These data suggest that the actomyosin system is required to coordinate the formation and release of attachment sites observed in a variety of other motility systems [41], including *Plasmodium* sporozoites [22, 34].

### Bead translocation does not depend on the actomyosin system

Apicomplexan parasites are known to translocate beads from the apical to the basal end and such ‘capping activity’ has been directly implicated in the motility of many cell types, including *Plasmodium* sporozoites [21]. Interestingly, treatment of sporozoites with CD had no significant effect on the speed of retrograde flow, while the force generated at the attached bead was reduced [21]. This could also be explained by the reduced formation of attachment sites, meaning a failure in force transmission rather than production. We therefore wished to analyse if retrograde membrane flow can occur in absence of the actomyosin system.

**Fig. 10:**
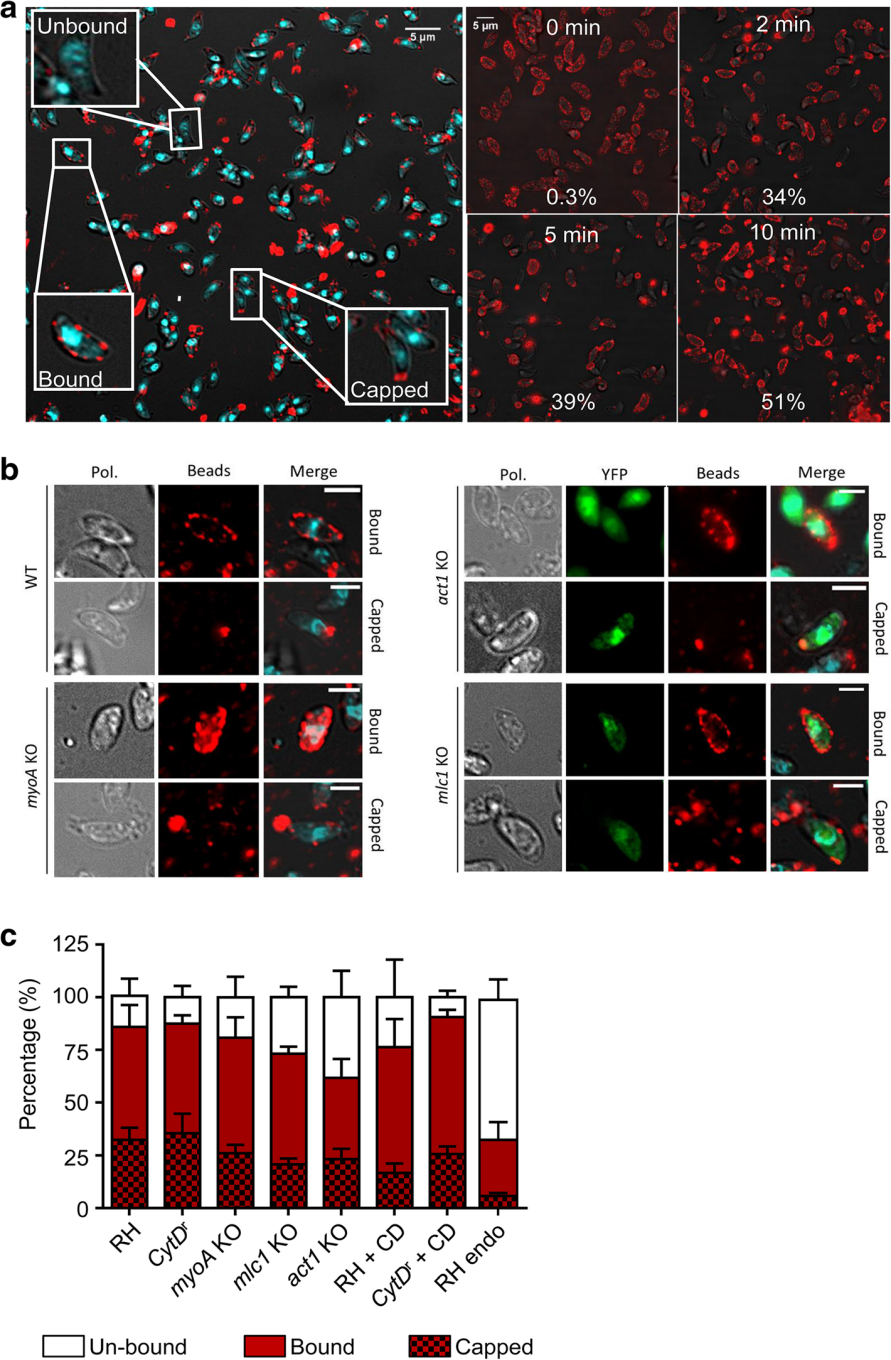
Analysis of bead translocation. **a** Three types of interactions of parasites with beads were observed (*left panel*); no interaction (unbound), beads located all around the parasite (bound), and beads translocated to the posterior end of the parasite (capped). Kinetics of bead translocation on wildtype parasites (RH) (0–10 min; *right panel*); the percentage of parasites showing a capped pattern at each time point is indicated. **b** Representative images of bead assays at 10 min in different genetic backgrounds, illustrating the binding and capping capacity of RH, *myoA* knockout (KO), *act1* conditional knockout (cKO) and *mlc1* cKO parasites. **c** The amount of bead binding and capping was quantified after 10 min incubation in different parasite strains: un-bound (*white*), bound (*red*) and capped (*red/black* patterned). **c** RH, Cytochalasin-D (CD) resistant (CytD^r^), *myoA* KO, *mlc1* cKO, *act1* cKO parasites, and RH incubated in Endo-buffer (RH Endo). Capping in the presence of 0.5 μM CD was also evaluated for wildtype and CytD^r^ parasites. The percentage of bead interaction with the parasites’ surface was significantly reduced in the RH Endo and *act1* cKO parasites. Capping still occurs in all KO mutant strains. Results are representative of at least three independent experiments ± standard deviation with at least 1000 parasites counted per experiment, per condition (*n* = 3)

We modified a bead translocation assay first developed by King et al. over 30 years ago [44]. Incubation of parasites with latex nano-beads leads to efficient binding of beads along the surface of the parasites and, within 10 min, 51% of parasites translocated beads to the parasite’s basal end (Fig. 10a). We next tested the role of the actomyosin system in the process of bead translocation, using either pharmacological perturbation or parasite mutants. In these experiments, we classified parasites as either ‘unbound’, ‘bound-uncapped’ or ‘capped’ (Fig. 10a). When parasites were kept in Endo buffer, which is known to diminish microneme secretion and motility [45], we found that a significant number of parasites were incapable of binding and translocating beads (Fig. 10c). When we tested the role of the actomyosin system in bead translocation (Fig. 10b, c), no abrogation of bead translocation was observed, suggesting the presence of retrograde flow in absence of the actomyosin system. Interestingly, in the *mlc1* cKO, *myoA* KO or parasites treated with CD, fewer beads appeared to bind to the parasite surface (Fig. 10c), confirming a role of the actomyosin system in attachment. Nevertheless, capping in the *act1* cKO occurred normally compared to RH when we assume that capped beads must have firstly bound. Similarly, the addition of CD had only minor effects on capping in the wt parasites. Overall, this demonstrates that bead translocation, and therefore the establishment of retrograde membrane flow, can occur independently of the actomyosin system of the parasite.

## Discussion

In this study, we set out to reconcile currently conflicting interpretations regarding the role of the apicomplexan actomyosin system during gliding motility and host cell invasion [1]. While there is no doubt about a critical role of the actomyosin system during motility and invasion, alternative models and mechanisms regarding the exact function of this system have been put forward based on biophysical, reverse genetic and inhibitor studies [3, 21, 22, 26, 38]. Indeed, several observations cannot be easily reconciled with the canonical linear motor model: (1) Biophysical studies on malaria sporozoites demonstrate the discrete, localised turnover of attachment sites that are not evenly translocated along the surface of the parasite [22]. Adhesion sites are formed/disengaged at the front and rear ends of the zoite, while the sporozoite undergoes a stretching phase. Interestingly, actin is important for the definition and release of the attachment sites [23], raising the possibility that apicomplexan motility is similar to amoeboid-like crawling. (2) *Eimeria* sporozoites show different types of motility (bending and pivoting). While pivoting appeared substrate- and actin-dependent, bending showed resistance to the actin-disrupting drug Cytochalasin B [46]. (3) A recent study demonstrates that retrograde membrane flow of malaria sporozoites occurs even at relatively high concentrations of the actin-disrupting drug CD [21]. (4) Reverse genetic approaches demonstrated that parasites remain motile in the absence of molecules thought to be key components of the motor machinery, including parasite actin [3–6].

In the case of reverse genetic studies, other groups have suggested that the observed phenotypes are due either to the presence of redundancies within the repertoire of genes involved in motility and invasion [24] or to an unusually stable actin (encoded by a single copy gene [47]), which persists many days and parasite generations after gene excision and can polymerize via an unusual isodesmic process that does not require a critical monomer (G-actin) concentration [27, 48]. While isodesmic polymerisation has been demonstrated in vitro, its in vivo relevance is unclear [49]. Previous polymerisation studies used G-actin produced in heterologous protein expression systems. However, a recent study demonstrated that apicomplexan actin is not properly folded when heterologously expressed due to differences in the chaperonin T-complex [50]. Furthermore, a recent study suggests that *Toxoplasma* actin is capable of forming F-actin structures in vivo that depend on a critical monomer concentration and demonstrates that F-actin is involved in multiple essential processes during intracellular parasite growth such as daughter cell assembly, vacuole organisation and parasite egress [28]. Using a conditional knockout for *act1* it was demonstrated that no ACT1 filament formation can occur as early as 48 h after removal of *act1* [28]. Finally, although apicomplexans possess actin-like proteins, it is unlikely that they can compensate for ACT1 and form filaments [27, 51]. Here, we performed a careful quantification of actin levels at different time points in the *act1* conditional knockout and find that actin is still detectable at 48 h, but fully depleted by 96 h after induction with rapamycin.

In good agreement with previous studies [3, 29], we observed essential roles of actin in apicoplast segregation, host cell egress and dense granule motility upon deletion of *act1*, while parasite motility and host cell invasion still occur, albeit at significantly reduced levels. Since these findings were in direct conflict with a number of studies that use actin-modulating drugs to probe for actin function, we also performed an analysis of the effects of the actin-disrupting drugs CD, LatA, and LatB, and the actin-stabilising drug Jas on wt and *act1* cKO parasites. While the effect of Jas appears to be specific for actin, since treatment of *act1* cKO parasites showed no effect of this drug, we find that CD is not uniquely specific for actin, since similar effects on parasite motility can be observed irrespective if wt, *act1* cKO or parasites expressing ACT1^CDr^ were analysed. While CD is certainly acting on parasite actin, as indicated by the significant differences between wt and CytD^r^ parasites, we find that concentrations above 0.5 μM CD lead to significant, non-actin-dependent effects on parasite motility, confirming previous studies [33]. In contrast, latA and latB, previously used to analyse the role of apicomplexan actin [36], appear to have no effect on tachyzoite actin, as noted previously. Together, these data indicate great care should be taken in choosing the correct concentration for actin-modulating drugs, especially when using CD, where an independent, unknown target(s) appears to be present in *T. gondii* [32].

Though all myosins are thought to depend on F-actin to function as a motor and, therefore, the *act1* cKO likely reflects the cumulative phenotype of all myosins, we were interested in the extent of redundancy between MyoA and MyoC, since previous studies indicated that MyoC can complement a MyoA deficiency [3, 39]. While we confirm that MyoC can partially complement for MyoA function, this complementation appears to depend on overexpression and consequent localisation of MyoC at the IMC. Indeed, our localisation analysis of MyoC in wt and *myoA* KO parasites demonstrates that, under physiological conditions, no significant relocation of MyoC to the IMC can be detected. In contrast, strong overexpression leads to redistribution of MyoC to the IMC, as described previously [39]. This redistribution appears to be also critical for functional complementation, leading to a higher ratio of motile parasites and host cell invasion. However, MyoC cannot compensate for MyoA function completely, since the parasites overexpressing MyoC remain as slow as uncomplemented *myoA* KO parasites. These results are also in good agreement with a recent study demonstrating that MyoA is the central myosin involved in host cell invasion and that, in the absence of MyoA, host cell membrane dynamics play an important role in invasion [52]. Importantly, deletion of *mlc1* results in a complete loss of the motor complex at the IMC, therefore representing a functional double KO for MyoA and MLC1. This mutant can be kept in cell culture indefinitely when the strong egress phenotype is bypassed by artificially releasing the parasites, followed by enrichment of the population using FACS, demonstrating that MLC1 is not essential for motility and host cell invasion per se. Indeed, *mlc1* cKO parasites behave similarly to *act1* cKO regarding motility and attachment to the surface.

Together, this analysis suggests a role for the actomyosin system during parasite motility that is distinct from the canonical linear motor model. We hypothesise that, rather than being the force generator during motility, the actomyosin system is instead involved in the regulation of attachment sites that are required to transmit the motility force to the substrate. To address this hypothesis, we used the above mutants to analyse different parameters that are likely crucial for efficient parasite motility such as attachment strength and retrograde membrane flow. We found that most *act1* cKO and *mlc1* cKO parasites remain immobile. However, those that move do so at speeds similar to wt parasites, albeit with shorter trajectories, suggesting that the actomyosin system is required to initiate and maintain motility. In a 3D environment the motility defects are stronger than in a 2D environment, with most parasites showing a displacement of less than one parasite length (7 μm). In contrast, these mutants are virtually unable to attach, as measured under flow conditions, indicating an important role in the formation of attachment sites, as previously suggested [22]. Unlike the *mlc1* cKO and *act1* cKO, *myoA* KO parasites show strong attachment to the surface, but significantly reduced speeds that cannot be compensated by overexpressing MyoC. We interpret these data to indicate that actin is required to form attachment sites, whereas MyoA might be required for the regulated release of these sites, leading to efficient force transmission during motility. A role for myosins in regulating adhesion and thereby traction force has recently been demonstrated [53]. Here, the balance between myosin contraction and adhesion strength determines the frequency of motility initiation [53]. A similar regulation of myosin contraction might occur in the case of apicomplexans in order to initiate motility and failure to do so could result in reduced motility rates. In this scenario, how is the motility and invasive force produced by the parasite? Previous studies implicated the generation of a retrograde membrane flow in parasite motility [21]. Interestingly, the flow itself is relatively resistant to actin-modulating drugs. Here, we modified a bead translocation assay as an indicator of retrograde membrane flow [44] to analyse the role of ACT1, MLC1 and MyoA in establishing membrane flow. Surprisingly, while fewer beads bind to the surface of the parasite in the absence of these proteins, bead translocation itself appears to be not significantly affected, again indicating a role of the actomyosin system in the formation of attachment sites (in this case for latex beads), but not in bead translocation. Future experiments and tools will be required to carefully address the role of retrograde membrane flow in force production and its exact role, if any, during motility and invasion.

## Conclusion

In conclusion, our study demonstrates the importance of the actomyosin system during motility and invasion. It is worth noting that, in physiological settings, this system is absolutely essential for parasite viability. In fact, in vivo experiments with *myoA* KO parasites demonstrate the complete inability of these parasites to establish an infection or to induce an adaptive immune response (our unpublished results). However, it is important to understand the molecular functions of the components of the gliding and invasion machinery in order to obtain a clear picture of how the individual components of this fascinating system work together to allow the parasite to move, invade into and egress from the host cell.

## Methods

### Cloning DNA constructs

All primers used in this study are listed in Additional file 13: Table S1 and were synthesised from Eurofins (UK).

### LoxP*act1*^CDr^ geneswap vector

The *act1* ORF containing the mutation C408G at the nucleotide level which confers the amino acid mutation of A136G was synthesised by GenScript (USA). Both the original LoxPAct1 geneswap vector [4] and the synthesised plasmid were digested with *Xma*I and *Pac*I to replace *act1* cDNA.

### p5RT70-Ty-MyoC and pMyoC-Ty-MyoC

To generate p5RT70-Ty-MyoC, the *myoC* ORF was amplified from cDNA using the primers *myoC* ORF fw/rv. The fragment was cloned via *Avr*II and *Pac*I into the parental plasmid p5RT70-Ty-KillerRed. To put *myoC* under its endogenous promotor a 1 kb fragment upstream of the start codon of *myoC* was amplified from genomic DNA using oligos p*myoC* fw/rv and cloned using as template vector p5RT70-Ty-MyoC via *Kpn*I and *Avr*II.

### Endogenous tagged MyoC

This vector was generated by cloning two 5’UTR fragments obtained by amplifying genomic DNA and a fragment of *myoC* gene using pBSSK + pSag1-Ble-Sag1 as a template vector. Fragment 1, *pMyoC*, was obtained by amplification of a 1 kb fragment upstream of the start codon of *myoC*, via *Hpa*I-*Not*I sites using oligos p*myoC*_2_ fw/rv. Fragment 2, 5’UTR *myoC*, was obtained by amplifying a region of approximately 1.5 kb located 1 kb upstream of the *myoC* start codon. Amplification was done using primers 5’UTR *myoC* fw/rv and cloned via *Kpn*1-*Xho*I. Finally, a 2.2 kb fragment of *myoC* was amplified using primers *myoC* gene fw/rv with a Ty-tag included in the forward primer. Subsequently, cloning was performed using enzymes *Spe*I/*Not*I.

### pMyoA-Ty-MyoA

To express *myoA* under its endogenous promotor a 2 kb fragment upstream of the start codon of *myoA* was amplified from genomic DNA using oligos 5’UTR *myoA* fw/rv and cloned using as template vector p5RT70-Ty-MyoA via *Kpn*I and *Eco*RI.

### Culturing of parasites and host cells

Human foreskin fibroblasts (HFFs) (SRC-1041, ATCC®) were grown on tissue culture-treated plastics and maintained in Dulbecco’s modified Eagle’s medium (DMEM) supplemented with 10% foetal bovine serum, 2 mM L-glutamine and 25 mg/mL gentamycin. Parasites were cultured on HFFs and maintained at 37 °C and 5% CO_2_. Cultured cells and parasites were regularly screened against mycoplasma contamination using the LookOut® Mycoplasma detection kit (Sigma) and cured with Mycoplasma Removal Agent (Bio-Rad) if necessary.

### *T. gondii* transfection and selection

To generate stable expressing parasites, 1 × 10^7^ of freshly lysed RH *∆hxgprt* [54] or RH-DiCre ∆*ku80* [4] parasites were transfected with 20 μg DNA by AMAXA electroporation. Selection was based on mycophenolic acid and xanthine, as described previously [54], or bleomycin.

### Generation and verification of parasite lines

The conditional *act1* KO^CDr^ strain (RH-DiCre ∆*ku80/endogenous act1::loxPact1* ^*CDr*^ *loxP*, referred to as *loxPAct1* ^*CDr*^) was generated by replacing the *act1* cassette in the LoxPAct1 geneswap vector [3] with a synthesised *act1* cassette containing the mutation Ala 136 Gly [32]. Integration after transfection was confirmed by PCR using primer sets (1-1’) for *act1* ORF (gDNA vs. cDNA), 5’UTR integration and cre-mediated recombination with primer sets (2-2’) and 3’UTR integration (3-3’).

For stabilisation of *p5RT70-Ty-MyoC*, *pMyoC-Ty-MyoC* and *pMyoA-Ty-MyoA*, RH ∆*hxgprt* and *myoA* KO parasites were selected using bleomycin. The vector pBSSK + pSag1-Ble-Sag1 was co-transfected with each respective construct in the proportion (2:1), and selection was carried out as described previously [55].

### Inducing conditional knockout lines

*act1* cKO and *mlc1* cKO were obtained by addition of 50 nM and 100 nM of rapamycin to the parental lines, respectively. Strains were incubated for 4 h at 37 °C and 5% CO_2_, and cultured as described previously [3]. To increase the population of these lines since they have an egress phenotype, *act1* cKO and *mlc1* cKO parasite media was swapped for DMEM^complete^ supplemented with 2.5% dextran sulphate after 24 h to enrich egress mutants [56].

### Immunofluorescence analysis

Immunofluorescence analysis was carried out as previously described [3]. Briefly, parasites were allowed to invade and replicate in an HFF monolayer grown on glass coverslips. The intracellular parasites were fixed in 4% paraformaldehyde for 20 min at room temperature. Afterwards, coverslips were blocked and permeabilised in 2% BSA and 0.2% Triton X-100 in PBS for 20 min. The staining was performed using the indicated combinations of primary antibodies (Additional file 14: Table S2) for 1 h. Followed by the incubation with secondary AlexaFluor 350, AlexaFluor 488, AlexaFluor 594 or AlexaFluor 633 conjugated antibodies (1:3000, Invitrogen – Molecular Probes) for another 45 min, respectively.

### Structured illumination microscope (SIM) imaging

Super-resolution SIM was carried out using an ELYRA PS.1 microscope (Zeiss). Images were acquired using a Plan Apochromat 63×, 1.4 NA oil immersion lens, recorded with a CoolSNAP HQ camera (Photometrics), and analysed using ZEN Black software (Zeiss) and ImageJ software.

### Western blot

Extracellular parasites were pelleted and then resuspended in RIPA buffer (50 mM Tris-HCl pH 8; 150 mM NaCl; 1% Triton X-100; 0.5% sodium deoxycholate; 0.1% SDS; 1 mM EDTA), incubation for 5 min on ice was used to lyse the cells. Afterwards, samples were centrifuged for 60 min at 14,000 rpm at 4 °C and laemmli buffer was added to the supernatant. A total of 5 × 10^6^ parasites were loaded onto an SDS acrylamide gel while, for *act1* cKO down-regulation, only 1 × 10^5^ parasites were loaded. Western blotting was performed as described previously [57] using IRDye680RD or IRDye800RD (Li-Cor) secondary antibodies.

### Protein quantification

Proteins were detected using the Li-Cor Odyssey and quantified using infrared detection of the protein of interest on Image Studio 5.0 (Li-Cor).

### Quantitative immunofluorescence assay (IFA)

Wt and parental LoxP strains were used as controls. For knockout lines, parasites were induced with rapamycin as described previously [3] and cultured for 24 h on HFFs prior to fixation. An IFA was conducted using the respective primary antibody; TgACT1 (Soldati, 1:100) or TgMLC1 (Soldati, 1:2000) and AlexaFlour 594 (Molecular Probes, 1:3000) as the secondary antibody. Images were obtained using a DeltaVision® Core microscope equipped with a CoolSNAP HQ2 CCD camera and were saved as single red and green channel files. Freeware CellProfiler 2.1.1 software (www.cellprofiler.org) was used to analyse and quantify fluorescence intensities. Greyscale images were imported to the program for analysis. The pipeline was adjusted to detect objects within the range of 5 to 40 pixels. Objects were identified using a global threshold strategy with a three class-Otsu threshold and weighted variances. Under these parameters, we identified in each image YFP-expressing parasites (generated by the DiCre cassette after correct gene excision) and the red coloured region of interest (corresponding to ACT1 and MLC1, respectively). The red signal was quantified on the basis of the total pixel occupied by the YFP expressing objects. The intensity of the red channel was measured for each object and exported to an Excel spreadsheet. The data obtained was filtered manually using area and integrated intensity parameters to include all parasites in a vacuole and exclude clustered vacuoles or random objects. Intensities from 60 vacuoles per time point were quantified and processed to remove the calculated internal background. The background fluorescence of the host cells was calculated using a YFP+ strain that was allowed to replicate under the same conditions as the tested parasites. However, no antibodies were added to the coverslips and, from this, the auto-fluorescence of the host was calculated and plotted as a horizontal dotted line.

### Cell fluorescence measurement

An IFA was performed in *myoA* KO and RH Δ*Ku80* lines expressing MyoC under different levels (p5RT70-Ty-MyoC, pMyoc-Ty-MyoC and endogenous tagged MyoC). Primary antibody against Ty-tag was used to localise MyoC and DAPI as a DNA marker. Images were obtained using ELYRA PS.1 microscope (Zeiss). All 3D SIM images were collected at the same exposure and resolution parameters. Images were obtained using Zen Black (Zeiss) and analysed using ImageJ. Vacuoles to be analysed were visually identified and traced using the drawing tool. Measurements obtained were area, integrated density and mean grey value. Background measurements were obtained by selecting a portion of the image with no signal. Data was plotted by using the corrected total cell fluorescence (CTCF) which was calculated using the following formula: [CTFC = Integrated density – (Area of selected cell × Mean fluorescence of background readings)] [58].

### Phenotypic characterisations

#### Plaque assay

Was conducted as described previously [59]. Briefly, 1 × 10^3^ parasites were inoculated on a confluent layer of HFFs and incubated for 5 days, after which the HFFs were washed once with PBS and fixed with ice cold MeOH for 20 min. HFFs were stained with Giemsa with plaque area measured using Fiji software. Mean values of three independent experiments ± standard deviation were determined.

#### Trail deposition assay

Gliding assays were performed as described before [7]. Briefly, freshly lysed parasites were allowed to glide on FBS-coated glass slides for 30 min before they were fixed with 4% paraformaldehyde (PFA) and stained with α-SAG1 under non-permeabilising conditions. Mean values of three independent experiments ± standard error of the mean (SEM) were determined. Where actin-modulating drugs were used, parasites were pre-incubated for 10 min in the respective concentration before the start of the assay.

#### Invasion assay

For the assay, 5 × 10^4^ freshly lysed parasites were allowed to invade a confluent layer of HFFs for 1 h. Subsequently, five washing steps were performed for removal of extracellular parasites. Cells were then incubated for a further 24 h before fixation with 4% PFA. Afterwards, parasites were stained with the α-IMC1 antibody [3]. The number of vacuoles in 15 fields of view was counted. Mean values of three independent experiments ± SEM were determined.

#### Egress assay

Egress assays were performed as described previously [60]. Briefly, 5 × 10^4^ parasites were grown on HFF monolayers for 36 h. Media was exchanged for pre-warmed, serum-free DMEM supplemented with 2 μM A23187 (in DMSO) to artificially induce egress. After 5 min, the cells were fixed with 4% PFA and stained with a-SAG1 antibody; 200 vacuoles were counted for their ability to egress out of the host cells. Mean values of three independent assays ± SEM were determined.

### Fluorescence activated cell sorting (FACS)

Prior to sorting, *mlc1* cKO parasites were scratched, syringed through a 25-G needle and filtered through a 3-μm Millipore filter (Millipore Merck) for purification of the sample. An S3e™ Cell Sorter (Bio-Rad Laboratories, Inc.), equipped with 488, 561 and 640 nm lasers, was used to sort *mlc1* cKO/YFP expressing parasites. Parasites were sorted into 5 mL tubes. The temperature for sample loading stage and collection area was set at 37 °C for culturing parasites and at 4 °C for western blot analysis. Before sorting parasites, the equipment was calibrated using ProLine™ universal calibration beads (Bio-Rad Laboratories, Inc.). Gates were adjusted to separate and collect YFP expressing population (*mlc1* cKO). For the long-term culturing parasites experiment, sorting was performed in single cell mode in order to obtain the purest population possible. After sorting, parasites were cultured under standard conditions described above. For western blot analysis, sorting was performed in enrichment mode to obtain the highest number of parasites possible.

### 2D motility assay

Time-lapse video microscopy was used to analyse the kinetics over a 2D surface similarly to as previously described [3]. Briefly, Ibidi μ-dish^35mm-high^ were coated in 100% FBS for 2 h at room temperature. Freshly egressed parasites were added to the dish. Time-lapse videos were taken with a 20× objective at one frame per second using a DeltaVision® Core microscope. Analysis was made using ImageJ wrMTrckr tracking plugin. For analysis, 20 parasites were tracked during both helical and circular trails with the corresponding distance travelled, average and maximum speeds determined. Mean values of three independent experiments ± standard deviation were determined.

### Invasion movies

Parasites contained in heavily infected HFFs were scratched and passed through a 26-G needle three times to release parasites artificially. Parasites were added to HFFs grown in Ibidi m-Dish^35mm-high^. Invasion events were observed after approximately 5 min when parasites had settled and penetration time was determined. Time-lapse videos were recorded at 40× objective at a rate of one frame per second using DeltaVision® Core microscope. Invasion events and time were manually tracked and calculated.

### 3D motility assay

Tachyzoites were prepared and assayed as previously described [42]. Briefly, the rapamycin-induced populations were harvested by syringe release of infected HFF monolayers using a 27-G needle, filtered through a 3-μm Nuclepore filter, and gently centrifuged at 1000 × *g* for 4 min. The tachyzoite pellet was washed and resuspended at a final concentration of 1–2 × 10^8^ tachyzoites/mL in 3D Motility Media (1× Minimum Essential Medium lacking sodium bicarbonate, 1% (v/v) FBS, 10 mM HEPES pH 7.0 and 10 mM GlutaMAX L-alanyl-L-glutamine dipeptide) supplemented with 0.3 mg/mL Hoechst 33342. The tachyzoite suspension was then mixed with three volumes of 3D Motility Media and three volumes of Matrigel (BD Biosciences, San Jose, CA), pre-chilled on ice. Motility in Matrigel was imaged, tracked and processed using Imaris × 64 v. 7.6.1 software (Bitplane AG, Zurich, Switzerland), as previously described [42]. Three independent biological replicates, each with three technical replicates, were performed. Parameters calculated from 3D motility assays were analysed using two-way ANOVA with Sidak’s multiple comparisons test and with the Kolmogorov–Smirnov test using GraphPad Prism v. 6.01 (La Jolla, CA). Under our imaging conditions, trajectory displacements less than 2 μm (i.e. slightly less than one-third the body length of a parasite (~7 μm)) cannot be distinguished from Brownian motion as measured empirically with heat-killed parasites [42], and so a cut-off of 2 μm was applied to exclude stationary parasites. Longer displacements were considered parasite-driven and used for more detailed analysis of motility parameters.

### Bead assays

Ibidi live cell dishes (29 mm) were coated with 0.1% poly-L-lysine for 30 min and washed with MilliQ water. Fluorescent latex beads (FluoSpheres®, 0.04 μm, Invitrogen) were diluted with 5 μL in 400 μL Hanks Balanced Salt Solution + HEPES (25 mM) (described hereafter as H-H buffer) and sonicated twice for 2 min. After a short spin (10 s, 6000 × *g*), the supernatant was recovered and left on ice for 30 min before use. Parasites of interest were harvested (by scratch, syringe and filtration). Parasites were pelleted for 5 min at 3000 × *g*, washed and resuspended in cold 250 μL H-H buffer to achieve 10^7^ parasites/mL. Parasites were then transferred to poly-L-lysine-coated dishes and left on ice for 20 min; 5 μL of diluted beads were added to 250 μL of H-H buffer and added to the parasites. Immediately, the dish was incubated at 37 °C for 15 min. The experiment was stopped by addition of 2 mL of 4% PFA and incubated at 4 °C for 10 min. The PFA was washed gently and parasite nuclei stained with Hoechst 0.01%. For time course, the parasites were fixed at different time points after the addition of the beads. For the drug and buffer assays, parasites were incubated for 10 min in the buffer of interest before their incubation on the coated dish. Bead dilution and the rest of the experiment were also made using the same buffer (Endo Buffer or H-H buffer + 0.5 μM CD).

For each experiment (n), an average of 1000 parasites were analysed. Total numbers of parasites, number of parasites without beads, with beads bound and with beads capped were quantified (e.g. For RH n1, a total of 1615 parasites were counted, within those, 63 were not interacting with the beads, 1062 had beads around them and 490 had capped beads). For each condition, experiments were performed in three independent experiments *n* = 3. Mean values of each interaction type for three independent experiments ± SEM were determined and compared to RH.

### Attachment under fluidic shear stress

LoxP strains were induced to generate KOs as previously described in Egarter et al. [3]. Fresh extracellular parasites (4 × 10^5^ in total, consisting of approximately equal numbers of control and KO) were loaded into collagen IV coated fluidic chambers (Ibidi IB-80192) and allowed to attach at 37 °C for 20 min. PBS was pumped through the chamber using an ‘open loop flow’ microfluidic pump (KD Scientific Legato 200 syringe pump) system, similar to that described previously [61], to control flow rates and generate fluidic shear stress. In our setup, a flow rate of 1 mL/min achieves 3 dyn/cm^2^ shear stress at the surface of the channel. Flow at 0.1 mL/min (equivalent to 0.3 dyn/cm^2^) was used to remove all non-attached parasites. At each fluidic shear stress level, control and mutant parasites were counted from five fields of view per experiment. Parasite count after the 0.1 mL/min wash was taken as 100% of attached parasites. Counts at all other rates of flow were normalised to the 100%. Data collected was analysed using Excel to assess the significance of differences between control and mutants using Student t-test and further analysed using GraphPad Prism v. 6.01 software to display data as trends. Parasites in the chamber were monitored via a Zeiss Axio Vert.A1 microscope setup with a 40× objective combined with an AxioCam ICm1 camera and Zen capture software.

## Additional files

Additional file 1: Figure S1. Investigating various actin antibodies for specificity. The specificity of four antibodies raised against TgACT1 and two against PfACT1 were compared using western blotting and immunofluorescence assay (IFA). Commercially available actin antibodies were also tested; anti-β-actin (Sigma) and ACTN05(C4) (Abcam). (A) Immunoblot analysis is evaluating the specificity of the antibodies to actin from either host (HFF) or *Toxoplasma* cell lysates. All apicomplexan antibodies are specific to *Toxoplasma* actin whereas the commercial antibodies label both HFF and parasite actin at 42 kDa. (B) IFA of *act1* cKO parasites at 4 days after induction. (C) IFA analysis of *act1* cKO parasites at 8 days after induction. Some antibodies show persistent signal that is still present up to 8 days post-induction, when western blot or IFA with other antibodies show actin has dropped below detectable levels. Scale bars: 10 μm. (TIF 1787 kb)
Additional file 12: Figure S2. Analysis of trajectory displacements in 3D motility assays. (A) Histogram of the trajectory displacements for LoxPMyoA (green), *myoA* KO (red) and p5RT70-Ty-MyoC (blue) parasites. The data have been grouped into 2 μm bins, with the number on the x-axis denoting the centre of the bin. Inset shows an expanded view of all trajectory displacements > 25 μm. The distributions show clearly that no *myoA* KO parasites move with a final displacement of more than one parasite body length (~7 μm) and that the MyoC complemented line does not fully restore the longer displacement trajectories seen in the LoxPMyoA parasites. Kolmogorov–Smirnov test, D = 0.3475 for LoxPMyoA vs. *myoA* KO, D = 0.3919 for *myoA* KO vs. MyoC complemented, and D = 0.1027 for LoxPMyoA vs. MyoC complemented, with all *P* values < 0.0001. Note that the 0–2 μm bin (i.e. non-motile parasites) has been omitted in order to facilitate a better display of trajectory displacements of motile parasites. However, both non-motile and motile parasites were included in the Kolmogorov–Smirnov tests for statistical significance and in calculating percent of total on the plot. (B, C) The trajectory displacement distributions for LoxPMlc1 vs. *mlc1* cKO (B) and LoxPAct1 vs. *act1* cKO (C) were compared and plotted as described in panel A. Kolmogorov–Smirnov test, D = 0.3694 for LoxPMlc1 vs. *mlc1* KO, and D = 0.354 for LoxPAct1 vs. *act1* cKO, with both *P* values < 0.0001. (TIFF 1790 kb)
Additional file 13: Table S1. Oligonucleotides used in this study. (DOCX 15 kb)
Additional file 14: Table S2. Summary of primary antibodies used in this study. (DOCX 17 kb)

## References

[1] Meissner M, Ferguson DJ, Frischknecht F (2013). Invasion factors of apicomplexan parasites: essential or redundant?. Curr Opin Microbiol.

[2] Soldati D, Meissner M (2004). Toxoplasma as a novel system for motility. Curr Opin Cell Biol.

[3] Egarter S, Andenmatten N, Jackson AJ, Whitelaw JA, Pall G, Black JA (2014). The toxoplasma acto-myoA motor complex is important but not essential for gliding motility and host cell invasion. PLoS One.

[4] Andenmatten N, Egarter S, Jackson AJ, Jullien N, Herman JP, Meissner M (2013). Conditional genome engineering in Toxoplasma gondii uncovers alternative invasion mechanisms. Nat Methods.

[5] Rugarabamu G, Marq JB, Guerin A, Lebrun M, Soldati-Favre D (2015). Distinct contribution of Toxoplasma gondii rhomboid proteases 4 and 5 to micronemal protein protease 1 activity during invasion. Mol Microbiol.

[6] Shen B, Buguliskis JS, Lee TD, Sibley LD (2014). Functional analysis of rhomboid proteases during Toxoplasma invasion. MBio.

[7] Hakansson S, Morisaki H, Heuser J, Sibley LD (1999). Time-lapse video microscopy of gliding motility in Toxoplasma gondii reveals a novel, biphasic mechanism of cell locomotion. Mol Biol Cell.

[8] Bretscher MS (1996). Getting membrane flow and the cytoskeleton to cooperate in moving cells. Cell.

[9] Jimenez-Ruiz E, Morlon-Guyot J, Daher W, Meissner M (2016). Vacuolar protein sorting mechanisms in apicomplexan parasites. Mol Biochem Parasitol.

[10] Keren K (2011). Cell motility: the integrating role of the plasma membrane. Eur Biophys J.

[11] Cramer LP (2013). Mechanism of cell rear retraction in migrating cells. Curr Opin Cell Biol.

[12] Havrylenko S, Mezanges X, Batchelder E, Plastino J (2014). Extending the molecular clutch beyond actin-based cell motility. New J Phys.

[13] Stroka KM, Jiang H, Chen SH, Tong Z, Wirtz D, Sun SX (2014). Water permeation drives tumor cell migration in confined microenvironments. Cell.

[14] Friedl P, Alexander S (2011). Cancer invasion and the microenvironment: plasticity and reciprocity. Cell.

[15] Lammermann T, Bader BL, Monkley SJ, Worbs T, Wedlich-Soldner R, Hirsch K (2008). Rapid leukocyte migration by integrin-independent flowing and squeezing. Nature.

[16] Barry NP, Bretscher MS (2010). Dictyostelium amoebae and neutrophils can swim. Proc Natl Acad Sci U S A.

[17] Howe JD, Barry NP, Bretscher MS (2010). How do amoebae swim and crawl?. PLoS One.

[18] Bretscher MS (2014). Asymmetry of single cells and where that leads. Annu Rev Biochem.

[19] Maritzen T, Schachtner H, Legler DF (2015). On the move: endocytic trafficking in cell migration. Cell Mol Life Sci.

[20] Fogelson B, Mogilner A (2014). Computational estimates of membrane flow and tension gradient in motile cells. PLoS One.

[21] Quadt KA, Streichfuss M, Moreau CA, Spatz JP, Frischknecht F (2016). Coupling of retrograde flow to force production during malaria parasite migration. ACS Nano.

[22] Munter S, Sabass B, Selhuber-Unkel C, Kudryashev M, Hegge S, Engel U (2009). Plasmodium sporozoite motility is modulated by the turnover of discrete adhesion sites. Cell Host Microbe.

[23] Hellmann JK, Perschmann N, Spatz JP, Frischknecht F (2013). Tunable substrates unveil chemical complementation of a genetic cell migration defect. Adv Healthc Mater.

[24] Frenal K, Soldati-Favre D (2015). Plasticity and redundancy in proteins important for toxoplasma invasion. PLoS Pathog.

[25] Frenal K, Polonais V, Marq JB, Stratmann R, Limenitakis J, Soldati-Favre D (2010). Functional dissection of the apicomplexan glideosome molecular architecture. Cell Host Microbe.

[26] Tardieux I, Baum J (2016). Reassessing the mechanics of parasite motility and host-cell invasion. J Cell Biol.

[27] Drewry LL, Sibley LD (2015). Toxoplasma actin is required for efficient host cell invasion. MBio.

[28] Periz J, Whitelaw J, Harding C, Lemgruber L, Gras S, Reimer M, et al. Toxoplasma gondii establishes an extensive filamentous network consisting of stable F-actin during replication. bioRxiv. 2016. doi:https://doi.org/10.1101/066522.

[29] Heaslip AT, Nelson SR, Warshaw DM (2016). Dense granule trafficking in Toxoplasma gondii requires a unique class 27 myosin and actin filaments. Mol Biol Cell.

[30] Jacot D, Daher W, Soldati-Favre D (2013). Toxoplasma gondii myosin F, an essential motor for centrosomes positioning and apicoplast inheritance. Embo J.

[31] Shaw MK, Compton HL, Roos DS, Tilney LG (2000). Microtubules, but not actin filaments, drive daughter cell budding and cell division in Toxoplasma gondii. J Cell Sci.

[32] Dobrowolski JM, Sibley LD (1996). Toxoplasma invasion of mammalian cells is powered by the actin cytoskeleton of the parasite. Cell.

[33] Gonzalez V, Combe A, David V, Malmquist NA, Delorme V, Leroy C (2009). Host cell entry by apicomplexa parasites requires actin polymerization in the host cell. Cell Host Microbe.

[34] Hegge S, Munter S, Steinbuchel M, Heiss K, Engel U, Matuschewski K (2010). Multistep adhesion of Plasmodium sporozoites. Faseb J.

[35] Wetzel DM, Hakansson S, Hu K, Roos D, Sibley LD (2003). Actin filament polymerization regulates gliding motility by apicomplexan parasites. Mol Biol Cell.

[36] Wetzel DM, Schmidt J, Kuhlenschmidt MS, Dubey JP, Sibley LD (2005). Gliding motility leads to active cellular invasion by Cryptosporidium parvum sporozoites. Infect Immun.

[37] Hegge S, Uhrig K, Streichfuss M, Kynast-Wolf G, Matuschewski K, Spatz JP (2012). Direct manipulation of malaria parasites with optical tweezers reveals distinct functions of Plasmodium surface proteins. ACS Nano.

[38] Vahokoski J, Bhargav SP, Desfosses A, Andreadaki M, Kumpula EP, Martinez SM (2014). Structural differences explain diverse functions of Plasmodium actins. PLoS Pathog.

[39] Frenal K, Marq JB, Jacot D, Polonais V, Soldati-Favre D (2014). Plasticity between MyoC- and MyoA-glideosomes: an example of functional compensation in Toxoplasma gondii invasion. PLoS Pathog.

[40] Soldati D, Boothroyd JC (1995). A selector of transcription initiation in the protozoan parasite Toxoplasma gondii. Mol Cell Biol.

[41] Iwamoto DV, Calderwood DA (2015). Regulation of integrin-mediated adhesions. Curr Opin Cell Biol.

[42] Leung JM, Rould MA, Konradt C, Hunter CA, Ward GE (2014). Disruption of TgPHIL1 alters specific parameters of Toxoplasma gondii motility measured in a quantitative, three-dimensional live motility assay. PLoS One.

[43] Papaioannou TG, Stefanadis C (2005). Vascular wall shear stress: basic principles and methods. Hellenic J Cardiol.

[44] King CA (1981). Cell surface interaction of the protozoan Gregarina with concanavalin A beads - implications for models of gregarine gliding. Cell Biol Int Rep.

[45] Kafsack BF, Beckers C, Carruthers VB (2004). Synchronous invasion of host cells by Toxoplasma gondii. Mol Biochem Parasitol.

[46] Russell DG, Sinden RE (1981). The role of the cytoskeleton in the motility of coccidian sporozoites. J Cell Sci.

[47] Dobrowolski JM, Niesman IR, Sibley LD (1997). Actin in the parasite Toxoplasma gondii is encoded by a single copy gene, ACT1 and exists primarily in a globular form. Cell Motil Cytoskeleton.

[48] Skillman KM, Ma CI, Fremont DH, Diraviyam K, Cooper JA, Sept D (2013). The unusual dynamics of parasite actin result from isodesmic polymerization. Nat Commun.

[49] Kumpula EP, Kursula I (2015). Towards a molecular understanding of the apicomplexan actin motor: on a road to novel targets for malaria remedies?. Acta Crystallogr F Struct Biol Commun.

[50] Olshina MA, Baumann H, Willison KR, Baum J (2016). Plasmodium actin is incompletely folded by heterologous protein-folding machinery and likely requires the native Plasmodium chaperonin complex to enter a mature functional state. Faseb J.

[51] Gordon JL, Sibley LD (2005). Comparative genome analysis reveals a conserved family of actin-like proteins in apicomplexan parasites. BMC Genomics.

[52] Bichet M, Touquet B, Gonzalez V, Florent I, Meissner M, Tardieux I (2016). Genetic impairment of parasite myosin motors uncovers the contribution of host cell membrane dynamics to Toxoplasma invasion forces. BMC Biol.

[53] Barnhart E, Lee KC, Allen GM, Theriot JA, Mogilner A (2015). Balance between cell-substrate adhesion and myosin contraction determines the frequency of motility initiation in fish keratocytes. Proc Natl Acad Sci U S A.

[54] Donald RG, Carter D, Ullman B, Roos DS (1996). Insertional tagging, cloning, and expression of the Toxoplasma gondii hypoxanthine-xanthine-guanine phosphoribosyltransferase gene. Use as a selectable marker for stable transformation. J Biol Chem.

[55] Messina M, Niesman I, Mercier C, Sibley LD (1995). Stable DNA transformation of Toxoplasma gondii using phleomycin selection. Gene.

[56] Coleman BI, Gubbels MJ. A genetic screen to isolate Toxoplasma gondii host-cell egress mutants. J Vis Exp. 2012;60. https://www.ncbi.nlm.nih.gov/pmc/articles/PMC3350636/. 10.3791/3807 PMC3350636 22349295

[57] Herm-Gotz A, Agop-Nersesian C, Munter S, Grimley JS, Wandless TJ, Frischknecht F (2007). Rapid control of protein level in the apicomplexan Toxoplasma gondii. Nat Methods.

[58] Potapova TA, Sivakumar S, Flynn JN, Li R, Gorbsky GJ (2012). Mitotic progression becomes irreversible in prometaphase and collapses when Wee1 and Cdc25 are inhibited. Mol Biol Cell.

[59] Meissner M, Schluter D, Soldati D (2002). Role of Toxoplasma gondii myosin A in powering parasite gliding and host cell invasion. Science.

[60] Black MW, Arrizabalaga G, Boothroyd JC (2000). Ionophore-resistant mutants of Toxoplasma gondii reveal host cell permeabilization as an early event in egress. Mol Cell Biol.

[61] Harker KS, Jivan E, McWhorter FY, Liu WF, Lodoen MB (2014). Shear forces enhance Toxoplasma gondii tachyzoite motility on vascular endothelium. MBio.

[62] Nishi M, Hu K, Murray JM, Roos DS (2008). Organellar dynamics during the cell cycle of Toxoplasma gondii. J Cell Sci.

