# Supplementary figures and images for: Surface attachment, promoted by the actomyosin system of Toxoplasma gondii is important for efficient gliding motility and invasion
Source: BMC Biol. 2017 Jan 18;15:1. doi: 10.1186/s12915-016-0343-5 (PMC5242020; doi:10.1186/s12915-016-0343-5)

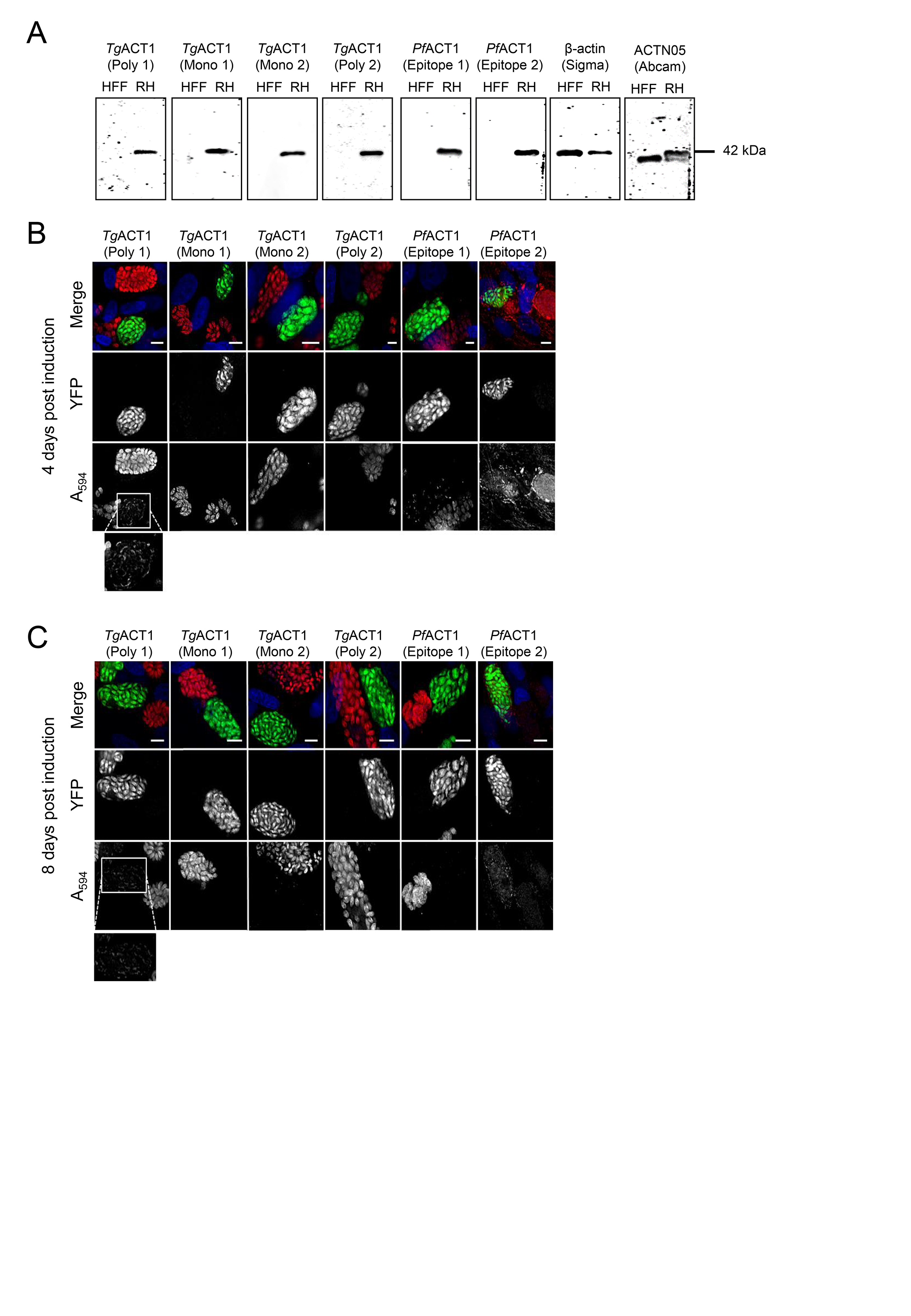

Supplement: Additional file 1: Figure S1. — Investigating various actin antibodies for specificity. The specificity of four antibodies raised against TgACT1 and two against PfACT1 were compared using western blotting and immunofluorescence assay (IFA). Commercially available actin antibodies were also tested; anti-β-actin (Sigma) and ACTN05(C4) (Abcam). (A) Immunoblot analysis is evaluating the specificity of the antibodies to actin from either host (HFF) or Toxoplasma cell lysates. All apicomplexan antibodies are specific to Toxoplasma actin whereas the commercial antibodies label both HFF and parasite actin at 42 kDa. (B) IFA of act1 cKO parasites at 4 days after induction. (C) IFA analysis of act1 cKO parasites at 8 days after induction. Some antibodies show persistent signal that is still present up to 8 days post-induction, when western blot or IFA with other antibodies show actin has dropped below detectable levels. Scale bars: 10 μm. (TIF 1787 kb) [file 12915_2016_343_MOESM1_ESM.tif]

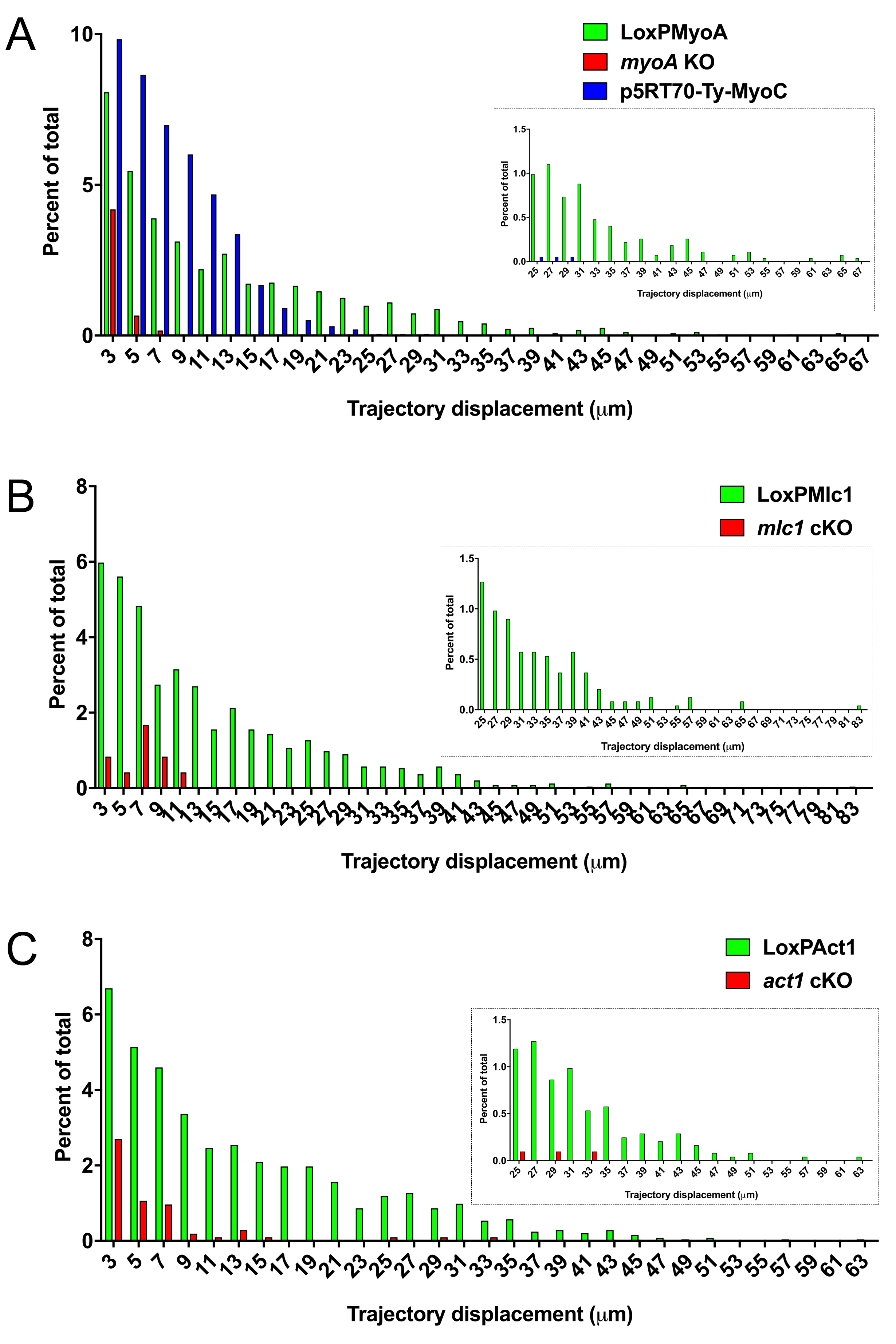

Supplement: Additional file 12: Figure S2. — Analysis of trajectory displacements in 3D motility assays. (A) Histogram of the trajectory displacements for LoxPMyoA (green), myoA KO (red) and p5RT70-Ty-MyoC (blue) parasites. The data have been grouped into 2 μm bins, with the number on the x-axis denoting the centre of the bin. Inset shows an expanded view of all trajectory displacements > 25 μm. The distributions show clearly that no myoA KO parasites move with a final displacement of more than one parasite body length (~7 μm) and that the MyoC complemented line does not fully restore the longer displacement trajectories seen in the LoxPMyoA parasites. Kolmogorov–Smirnov test, D = 0.3475 for LoxPMyoA vs. myoA KO, D = 0.3919 for myoA KO vs. MyoC complemented, and D = 0.1027 for LoxPMyoA vs. MyoC complemented, with all P values < 0.0001. Note that the 0–2 μm bin (i.e. non-motile parasites) has been omitted in order to facilitate a better display of trajectory displacements of motile parasites. However, both non-motile and motile parasites were included in the Kolmogorov–Smirnov tests for statistical significance and in calculating percent of total on the plot. (B, C) The trajectory displacement distributions for LoxPMlc1 vs. mlc1 cKO (B) and LoxPAct1 vs. act1 cKO (C) were compared and plotted as described in panel A. Kolmogorov–Smirnov test, D = 0.3694 for LoxPMlc1 vs. mlc1 KO, and D = 0.354 for LoxPAct1 vs. act1 cKO, with both P values < 0.0001. (TIFF 1790 kb) [file 12915_2016_343_MOESM12_ESM.tiff]
