# Supplementary material for: Surface attachment, promoted by the actomyosin system of Toxoplasma gondii is important for efficient gliding motility and invasion
Source: BMC Biol. 2017 Jan 18;15:1. doi: 10.1186/s12915-016-0343-5 (PMC5242020; doi:10.1186/s12915-016-0343-5)
Supplement: Additional file 13: Table S1. — Oligonucleotides used in this study. (DOCX 15 kb) [file 12915_2016_343_MOESM13_ESM.docx]

| **Table S1: Oligonucleotides used in this study** | | |
| --- | --- | --- |
| Name | Sequence 5’ → 3’ | Construct/Purpose |
| *myoC* ORF fw | GCCCTAGGATGGAGCGCAAACAAACCCAGATG | p5RT70-Ty-MyoC |
| *myoC* ORF rv | GCTTAATTAATCACGGTCTATCCGGCGCACAG | p5RT70-Ty-MyoC |
| p*myoC* fw | CCGGTACCTGCAACTCATGCACACGT | pMyoC-Ty-MyoC |
| p*myoC* rv | GGGCCTAGGGTCAAGTGGATCTTGGTTAGTATGGACCTCCATGGTGAAAAACAGGAAAGAAGGT | pMyoC-Ty-MyoC |
| p*myoC*_2_ fw | CCGTTAACTAGCTGGTGACGCTACCGCC | Endogenous tagging MyoC |
| p*MyoC*_2_ rv | CCGCGGCCGCaaACTAGTGTCAAGTGGATCTTGGTTAGTATGGACCTCCATGGTGAAAAACAGGAAAG | Endogenous tagging MyoC |
| 5’UTR*myoC* fw | CGGGTACCGCTTCACTGGTTTATCGTGTCACGG | Endogenous tagging MyoC |
| 5’UTR*myoC* rv | GGCTCGAGGGCGGTAGCGTCACCAGCTAG | Endogenous tagging MyoC |
| *myoC*gene frw | GCACTAGTATGGAGCGCAAACAAACCCAGATGATACTGG | Endogenous tagging MyoC |
| *myoC*gene rv | CCGCGGCCGCATCGATGAAGTGTTTTTGCAG | Endogenous tagging MyoC |
| 5’UTR*myoA* fw | GCACTAGTATGGAGCGCAAACAAACCCAGATGATACTGG | pmyoA-Ty-myoA |
| 5’UTR *myoA* rv | CCGCGGCCGCATCGATGAAGTGTTTTTGCAG | pmyoA-Ty-myoA |
| Act cDNA fw | GGGAATTCGACAAAATGGCGGATGAAGAAGTGCAAGCC | Int PCR 1-1’ |
| Act cDNA rv | CGTTAATTAAAAGCACTTGCGGTGGACGATGCTCGGG | Int PCR 1-1’ |
| Act1integ5' fw | CGTCACACCCGCTCAGCCAAAGG | Int PCR 2-2’ |
| YFP int rv | ATGGGCACCACCCCGG | Int PCR 2-2’ |
| \| HX fw \| GCACACCTGGTCTCGATGTCGAAC \| \| --- \| --- \| | GCTACGACTTCAACGAGATGTTCCGCG | Int PCR 3-3’ |
| Act1integ3' rv | GGCTCAAACATGACACGTGTGC | Int PCR 3-3’ |
|  |  |  |
