# Supplementary material for: Surface attachment, promoted by the actomyosin system of Toxoplasma gondii is important for efficient gliding motility and invasion
Source: BMC Biol. 2017 Jan 18;15:1. doi: 10.1186/s12915-016-0343-5 (PMC5242020; doi:10.1186/s12915-016-0343-5)
Supplement: Additional file 14: Table S2. — Summary of primary antibodies used in this study. (DOCX 17 kb) [file 12915_2016_343_MOESM14_ESM.docx]

| **Antibody** | **Literature Reference** | **Source** | **Commercial Reference** | **AB registry ID** |
| --- | --- | --- | --- | --- |
| βActin | (Tamura *et al*, 2014) | Sigma-Aldrich^®^ | (A2228) | AB_1008152 |
| ACTN05 (C4) | (Achanta *et al*, 2012) | Abcam | (ab3280) | AB_303668 |
| *Tg*Actin (Poly 1) | (Drewry & Sibley, 2015) |  |  |  |
| *Tg*Actin1(Mono1) | (Herm-Gotz *et al*., 2002) |  |  |  |
| *Tg*Actin1(Mono 2) | (Angrisano *et al*., 2012) |  |  |  |
| *Tg*Actin1(Poly 2) | (Angrisano *et al*., 2012) |  |  |  |
| *Pf*Actin1(Epitope 1) | (Zhang *et al*., 2011) |  |  |  |
| *Pf*Actin (Epitope 2) | (Zhang *et al*., 2011) |  |  |  |
| *Tg*Aldolase | (Starnes *et al.*, 2006) |  |  |  |
| *Tg*Catalase | (Ding *et al*, 2000) |  |  |  |
| *Tg*IMC1 | (Johnson *et al.*, 2007) |  |  |  |
| *Tg*MyoA | (Hettmann *et al*., 2000) |  |  |  |
| *Tg*MLC1 | (Herm-Gotz *et al*., 2002) |  |  |  |
| TyTag | (Bastin *et al*.,1996 ) |  |  |  |

**Table S2.** Summary of primary antibodies used in this study
